# Deciphering COVID-19 host transcriptomic complexity and variations for therapeutic discovery against new variants

**DOI:** 10.1016/j.isci.2022.105068

**Published:** 2022-09-03

**Authors:** Jing Xing, Rama Shankar, Meehyun Ko, Keke Zhang, Sulin Zhang, Aleksandra Drelich, Shreya Paithankar, Eugene Chekalin, Mei-Sze Chua, Surender Rajasekaran, Chien-Te Kent Tseng, Mingyue Zheng, Seungtaek Kim, Bin Chen

**Affiliations:** 1 Department of Pediatrics and Human Development, Michigan State University, Grand Rapids, MI 49503, USA; 2 Zoonotic Virus Laboratory, Institut Pasteur Korea, Seongnam-si, Gyeonggi-do, 13488, Korea; 3 Drug Discovery and Design Center, State Key Laboratory of Drug Research, Shanghai Institute of Materia Medica, Chinese Academy of Sciences, Shanghai 201203, China; 4 Departments of Microbiology and Immunology, The University of Texas Medical Branch, Galveston, TX 77555, USA; 5 Department of Surgery, Stanford University School of Medicine, Palo Alto, CA, USA; 6 Helen DeVos Children’s Hospital, Grand Rapids, MI 49503, USA; 7 Center of Biodefense and Emerging Infectious Diseases, The University of Texas Medical Branch, Galveston, TX 77555, USA; 8 Department of Pharmacology and Toxicology, Michigan State University, Grand Rapids, MI 49503, USA; 9 Department of Computer Science and Engineering, Michigan State University, East Lansing, MI 48824, USA

**Keywords:** Microbiology, Virology, Bioinformatics, Omics, Transcriptomics

## Abstract

The molecular manifestations of host cells responding to SARS-CoV-2 and its evolving variants of infection are vastly different across the studied models and conditions, imposing challenges for host-based antiviral drug discovery. Based on the postulation that antiviral drugs tend to reverse the global host gene expression induced by viral infection, we retrospectively evaluated hundreds of signatures derived from 1,700 published host transcriptomic profiles of SARS/MERS/SARS-CoV-2 infection using an iterative data-driven approach. A few of these signatures could be reversed by known anti-SARS-CoV-2 inhibitors, suggesting the potential of extrapolating the biology for new variant research. We discovered IMD-0354 as a promising candidate to reverse the signatures globally with nanomolar IC_50_ against SARS-CoV-2 and its five variants. IMD-0354 stimulated type I interferon antiviral response, inhibited viral entry, and down-regulated hijacked proteins. This study demonstrates that the conserved coronavirus signatures and the transcriptomic reversal approach that leverages polypharmacological effects could guide new variant therapeutic discovery.

## Highlights

• Robust coronavirus infection signatures were identified from 1,700 host profiles
• The signatures captured essential pathogenic biology of SARS-CoV-2 and its variants
• IMD-0354 reversed these signatures and inhibited new variants with nM IC50s
• The antiviral potential of IMD-0354 benefits from its polypharmacological effects

## Graphical abstract

**Figure:**
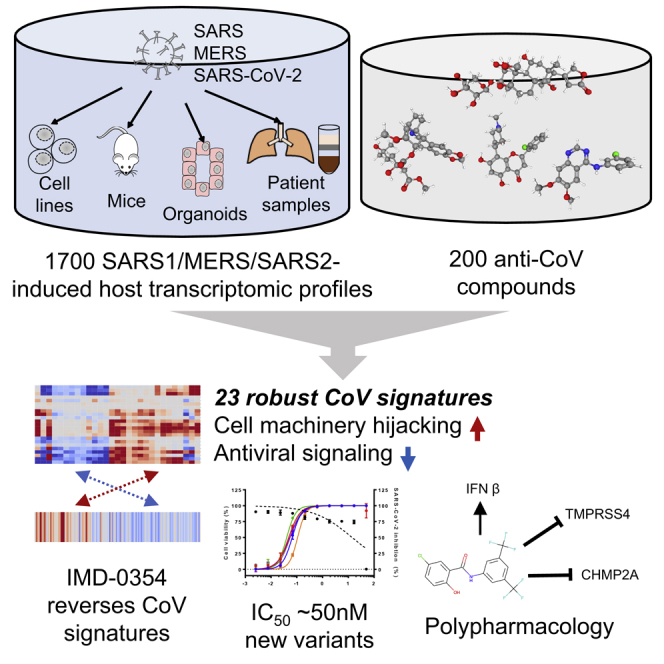

## Introduction

Since early December 2019, the newly emerged SARS-CoV-2 virus has infected more than 600 million people globally and claimed more than 6 million deaths (as of September 2022). In addition, severe COVID-19 has caused irreversible organ injuries in a large number of patients (Abbasi, 2021; Ankit et al., 2020; Nalbandian et al., 2021). The systems of host cells responding to SARS-CoV-2 infection under various models (cell lines, organoids, animals, and patients) and conditions (dosages, times, and patient severity) have been molecularly characterized using diverse omics profiling and perturbation technologies, often resulting in a list of candidate targets in individual studies (Blanco-Melo et al., 2020; Bojkova et al., 2020; Daniloski et al., 2020; Delorey et al., 2021; Demichev et al., 2021; Desai et al., 2020; McClain et al., 2021; Overmyer et al., 2021; Yang et al., 2021). Small molecules modulating these targets were subsequently proposed to treat COVID-19 (Chu et al., 2021; Samelson et al., 2022, p. 2). Together with phenotypic screening hits, more than 200 candidates surfaced in publications. However, few studies fully appreciated the complexity and variation of the virus-induced molecular changes in host cells (Dittmar et al., 2021; The COVID-19 Gene and Drug Set Library, 2020) and the prevalence of polypharmacological effect of small molecules (Lin et al., 2019), partially accounting for the moderate efficacy of repurposing candidates. Rapidly emerging variants still impose increasing threats because of their higher transmissibility, highlighting the pressing need of untangling the complexity and variation of transcriptional programs in host cells to aid the discovery of drugs for new variants and future pandemics.

**Figure 1:**
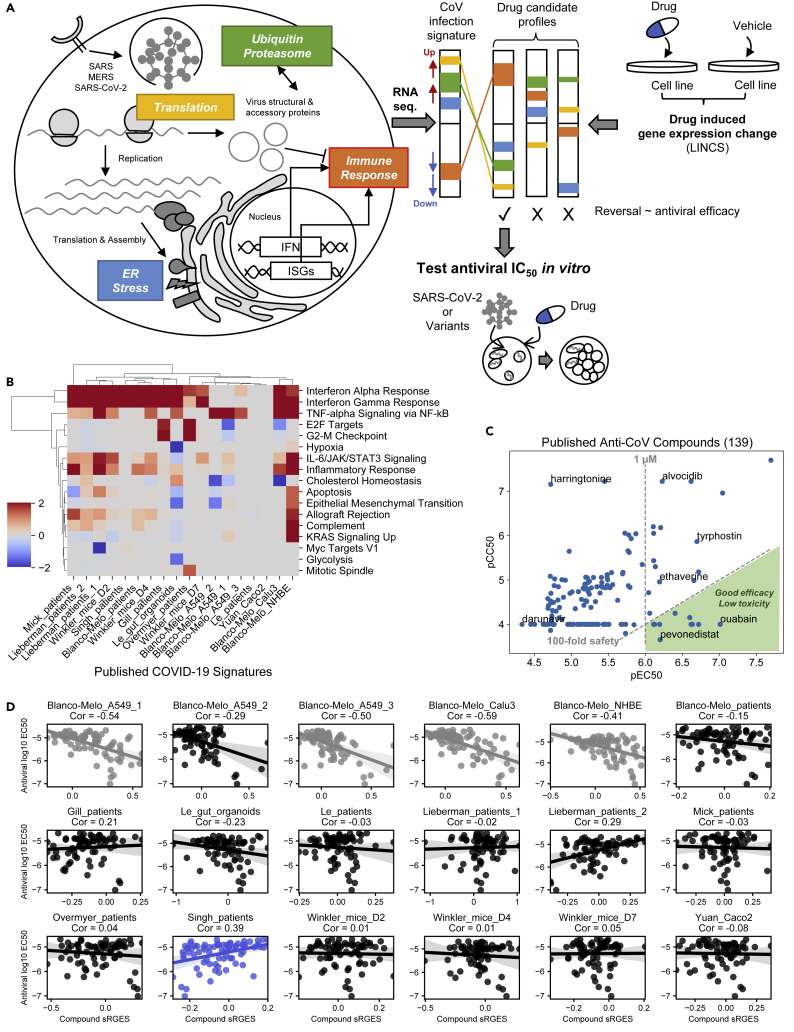
Systems-based drug discovery through targeting host transcriptional signature of CoV infection (A) Examples of biological processes involved in viral infection and the illustration of a drug-disease transcriptome reversal approach for drug discovery. LINCS: Library of Integrated Network-based Cellular Signatures. (B) Heatmap of enriched MSigDB Hallmark pathways in published COVID-19 signatures. Color code depicts p values (Fisher exact test, log10 transformed, inversed for up-regulation), with red representing up-regulation and blue representing down-regulation. (C) Summary of the efficacy and cytotoxicity of published *in vitro* anti-CoV hits. pEC50: -log10 EC_50_ in Molar; pCC50: -log10 CC_50_ in Molar. (D) The scatter plot and correlation of compound anti-CoV efficacies and CoV signature reversal scores. The signature names and Spearman coefficients are shown above the plots. Grey: negative correlation; black: not significant; blue: positive correlation. sRGES: summarized Reversal of Gene Expression Score. See also Table S1 and Figure S1.

Recent studies revealed that published anti-SARS-CoV-2 drugs share similar transcriptional programs (Xing et al., 2021) and 16 viruses tend to present conserved transcriptional regulation modules of immune responses (Zheng et al., 2021), suggesting the prevalence of underlying molecular mechanisms connecting antivirals and host responses. In cancer, reversal of transcriptional expression correlates with drug efficacy (Chen et al., 2017). We thus first sought to answer if published anti-SARS-CoV-2 drugs tend to reverse the global gene expression of infected host cells as observed in cancer. The central idea of the reversal approach (or called systems-based approach) is that the antiviral drugs could suppress the over-expressed genes and activate the repressed genes, regardless of drug mechanisms and biological systems involved (Figure 1A). Next, we asked if the findings could guide the identification of a robust transcriptional program that could drive the discovery of drugs for new SARS-CoV-2 variants.

We first surveyed published COVID-19 host signatures and observed that most of them were not reversed by known anti-SARS-CoV-2 drugs. We then interrogated the biological processes implicated in the 374 raw coronavirus (CoV) signatures derived from 1,700 open host transcriptomic samples of SARS, MERS, and SARS-CoV-2 infection and found that only 6% of them could be reversed by anti-SARS-CoV-2 drugs, suggesting the challenge of direct deployment of these raw signatures without denoizing the signal. We proposed a novel data-driven approach to tease out a set of robust CoV signatures by distilling knowledge from compounds effective against other species in the coronaviridae. Reversal of these signatures identified a potent drug candidate, IKK2 Inhibitor V (IMD-0354), against SARS-CoV-2 and five variants with nanomolar IC_50_ *in vitro*. RNA-seq profiling and systems biology analysis further confirmed it reversed a global host transcriptional program through targeting multiple virus-altered pathways rather than interacting with the primary target IKK2.

## Results

### Reversal of published COVID-19 signatures correlates poorly with antiviral efficacy

We compiled 18 COVID-19 signatures from the supplementary materials of nine studies, which profiled the host transcriptomic changes in lung cell lines, organoids, mice, and patients with SARS-CoV-2 infection (Table S1). Pathway enrichment analysis suggested that most of the published signatures displayed induced interferon and inflammatory responses, whereas some showed diverse or even contradictory patterns (Figure 1B). For example, the cholesterol homeostasis genes are up-regulated in an organoid model but down-regulated in a Calu-3 cell line model (Figure 1B). In addition to model differences, varying biological conditions could drive the diversity, as supported by the separation of the signatures derived from the same mouse model under different days (Figure 1B).

Because these signatures were developed to answer specific biological questions, to verify if they could be adopted for the systems-based drug discovery (Figure 1A) we then sought to leverage approximately 200 anti-SARS-CoV-2 repurposing candidates identified through the collective efforts worldwide. Most of the hits showed moderate activity or toxicity (Martinez, 2021; Singh et al., 2020) (Figures 1C and S1A, and Data S1), indicating a need for drugs to arrest viral infection effectively and safely, especially for new variants. Of note, compounds selectively inhibiting viral proteins (e.g., 3CL) or viral entry receptors (e.g., *ACE2* and *TMPRSS2*) might not present transcriptomic reversal of the dysregulated genes in the infected cells. By mapping to a few published screening results (Brimacombe et al., 2020), 38 positive compounds in our collection were found active to inhibit 3CL or Spike-ACE2 binding (efficacy >50% and AC_50_< 50 μM, Data S1). However, their antiviral activities at the cellular level are not always consistent with the target inhibitory activities, suggesting that targets related to host transcriptional programs were involved. Besides, 92 compounds in our list exhibit no obvious inhibition of the three targets, indicating they might act on the host targets. Among all 18 published COVID-19 signatures, only one showed a significant positive correlation between compound antiviral EC_50_ values and the scores of CoV signature reversal (sRGES) (Figure 1D) and only five could enrich positive hits (Figure S1B). A few even displayed a negative correlation and a negative enrichment (Figures 1D and S1B), meaning these signatures could lead to a hit rate even lower than random selection. This initial survey of published signatures suggests their considerable variation and a mixed-signal of informing drug discovery, thus a systematic investigation of all published transcriptome profiles is needed.

### A data-driven pipeline to generate robust CoV signatures from rich transcriptomic samples and antiviral drug profiles

To select valid disease signatures that capture the pathological biology of CoV infection, we developed a data-driven pipeline utilizing known antiviral compound-induced transcriptomic profiles. Of note, for emerging crises caused by SARS-CoV-2 and its variants, the infected host profiles and active drugs are not often readily available; thus, we aim for a robust model trained from known virus variants data that could be extrapolated to respond to future variants. For example, existing host gene expression profiles of samples infected by SARS-CoV or MERS-CoV might approximate those infected by SARS-CoV-2 based on their high genomic similarity (Lu et al., 2020). The high correlation of *in vitro* drug efficacy between anti-SARS-CoV and anti-MERS-CoV (Spearman correlation coefficient: 0.6, Figure S1C) further confirmed that drugs active against SARS-CoV or MERS-CoV might provide knowledge in SARS-CoV-2 drug screening.

**Figure 2:**
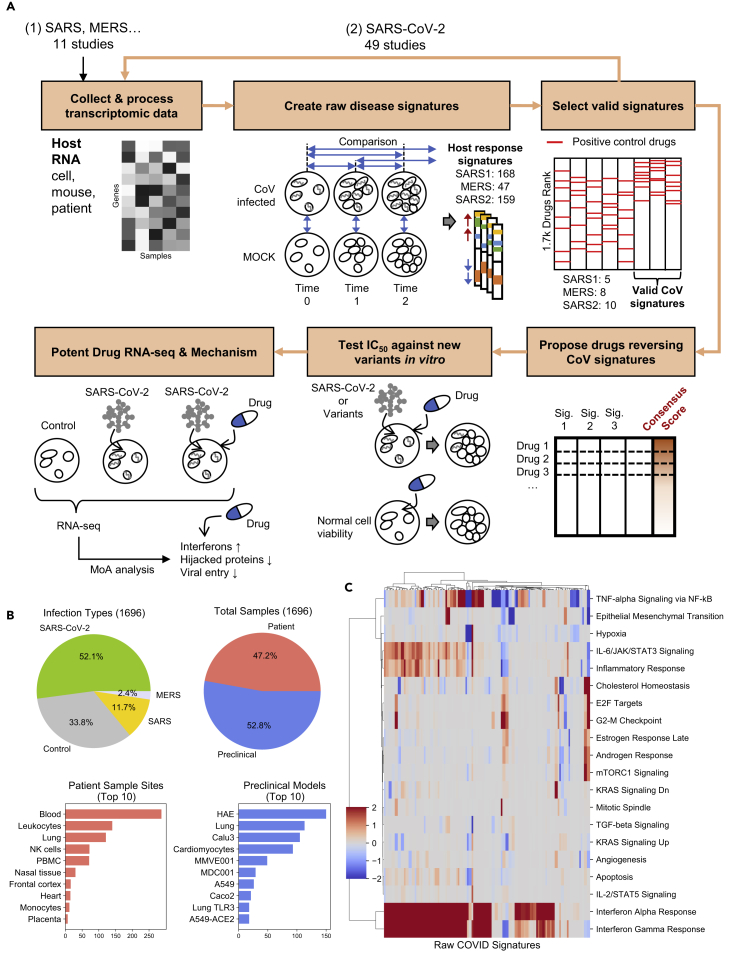
A pipeline of identifying robust CoV signatures and predicting new anti-CoV drugs (A) Workflow of infection signature identification from hundreds of transcriptomic profiles, drug antiviral effect evaluation, and mechanism interpretation. CoV: coronavirus. MoA: mechanism of action. (B) Summary of CoV-induced host transcriptomic change datasets, including different viruses, models, and patient sample categories. (C) Heatmap of enriched MSigDB Hallmark pathways in each raw CoV signature. Color darkness depicts p-values (Fisher’s exact test, log10 transformed, inversed for up-regulation), with red representing up-regulation and blue representing down-regulation. See also Figure S2.

In this pipeline (Figure 2A), we first processed microarray and RNA-seq data from different published studies of SARS-, MERS- and SARS-CoV-2-induced host gene expression change, including both preclinical models and COVID-19 patient samples, which generated various comparisons between infection and control or different infection stages. Based on their reversal pattern to the transcriptomic profiles of known small molecule CoV inhibitors, we defined valid CoV disease signatures, which were subsequently verified in multiple COVID-19 patient cohorts. Then a consensus prediction using valid CoV signatures was performed to propose potent drug candidates against SARS-CoV-2 and its variants of concern (VOC) followed by *in vitro* validation. Finally, we investigated its antiviral mechanism based on RNA-seq profiling and systems biology.

In total, 1696 host RNA-seq and microarray samples related to SARS/MERS/SARS-CoV-2 infection were collected from 57 published studies (Data S2). SARS-CoV-2 profiles account for more than half of all samples, much more than SARS and MERS samples (14%) because of the improved profiling technology and the greater impact of SARS-CoV-2 (Figure 2B). We considered COVID-19 patient samples (47.2%) spanning several severity levels (asymptomatic, mild, and severe, Figure S2A) and different tissue of origins (primarily from blood and lung) as well as preclinical (52.8%) samples including human cell lines (human airway epithelial (HAE) cells and other CoV susceptible cells like Calu-3), organoids, and mouse/ferret *in vivo* models (Figures 2B and S2B). An integrative analysis of these samples from diverse patient cohorts and experimental settings is deemed to provide a comprehensive picture of CoV-induced host responses. Again, most of the raw signatures implied over-activated interferon and inflammatory responses of the host (Figure 2C), which are natural defensive reactions for viral infection blockade and should not be suppressed by antiviral drugs. The patterns of other pathway enrichment are diverse across raw CoV signatures, corroborating with the survey of published signatures. For example, NF-kB, JAK/STAT, and cholesterol homeostasis could be both up- and down-regulated in different experiments. Together, reversing one or a few of the raw CoV signatures might not lead to a potent antiviral drug candidate.

To explore the potential of this pipeline in anti-CoV compound discovery, we evaluated its performance when (1) predicting SARS-CoV-2 inhibitors from SARS and MERS data only and (2) predicting external SARS-CoV-2 inhibitors from data of all three viruses.

**Figure 3:**
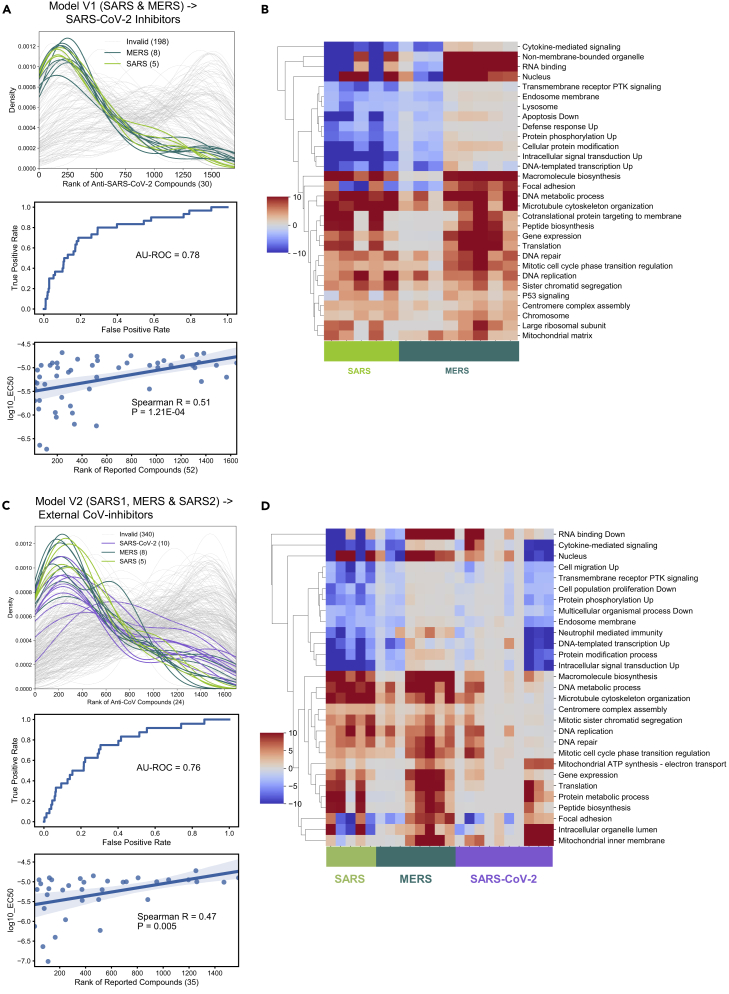
Valid CoV signatures and their performance in predicting CoV inhibitors (A) Top: Performance of five valid SARS signatures (green), eight valid MERS signatures (blue), and 198 invalid signatures (grey) evaluated with 30 anti-SARS-CoV-2 compounds. Middle: the receiver operating characteristic (ROC) curve of published SARS-CoV-2 inhibitors based on their predicted ranking in the entire drug profile library. Bottom: the correlation between log10 transformed EC_50_ (in Molar) and the predicted ranking of compounds with reported SARS-CoV-2 inhibitory effect. The validation dataset sizes are annotated in the X axis title. (B) Gene Ontology terms enriched across the valid CoV signatures v1. Color darkness depicts false discovery rate (FDR) (log10 transformed, inversed for up-regulation), with red representing up-regulation and blue representing down-regulation. (C) Performance of valid CoV Signatures v2 on an external positive compound set. (D) Gene Ontology terms enriched across the valid CoV signatures v2. See also Tables S2–S4, Figures S3–S5.

For the first task, 430 samples from public repositories, representing infections by MERS-CoV or SARS-CoV (and a few other strains for comparison) in different models (e.g., cell line and mouse models) across multiple time points were used to create raw disease signatures (Figure 2A and Table S2). Their expression profiles were generated using either microarray or RNA-seq. Data processing and signature creation methods were tailored for different profiling platforms (see STAR Methods). We enumerated all possible comparisons (Figure 2A), including (1) comparisons between infection and mock groups at each time point, and (2) comparisons between different time points within individual infection or mock groups (e.g., time point 1 versus time point 0, time point 2 versus time point 1). Each comparison resulted in a raw disease signature used to characterize the infection status, followed by calculating the sRGES of different drug transcriptomic profiles from the LINCS project (Subramanian et al., 2017). To evaluate the quality and pathologic relevance of each raw disease signature, we used positive drugs identified from *in vitro* MERS/SARS-CoV testing (38 positive drugs with known LINCS profiles, 29 with EC_50_ values; Figure S1C, Table S3 and Data S1). Among 215 MERS-CoV or SARS-CoV infection signatures, 13 signatures (i.e., robust CoV signature v1) were able to recover these positive control drugs (which were highly enriched at the top of the predicted drug lists; Figure S3, Data S3 and S4). Moreover, the EC_50_ of these drugs was also significantly correlated with sRGES (Figure S3). In contrast, we did not observe significant enrichment of positive control drugs using H1N1 infection signatures. We also confirmed the predictive power of this pipeline using 52 compounds with reported anti-SARS-CoV-2 *in vitro* efficacy (Data S1). Although derived from SARS and MERS profiles, each valid signature still enriched positive compounds (EC_50_< 10 μM) at the top, whereas the invalid signatures could not (Figure 3A). Also, the average rank could recall the published SARS-CoV-2 inhibitors with an AU-ROC of 0.78, and it correlated with reported anti-SARS-CoV-2 EC_50_ (Spearman R = 0.51, p = 1.21E-04) (Figure 3A). For prospective validation, a drug repurposing library including 1720 bioactive compounds with their LINCS profiles was screened against the valid CoV signatures for COVID-19 drug discovery based on a consensus score of the median rank of each drug across different CoV signatures (Data S5). In this pilot screening, seven out of ten compounds proposed by this pipeline prevented the SARS-CoV-2-induced cytopathic effect (CPE) within 10 μM (Table S4). These results indicated that the selected SARS and MERS signatures capture the essential CoV pathological biology and the strategy of reversing them is applicable for drug discovery against COVID-19 caused by SARS-CoV-2.

Gene Ontology (GO) enrichment analysis suggested common pathways among the 13 valid CoV signatures (Figure 3B, Data S6). Host regular activities that were inhibited by CoV infection include protein phosphorylation and modification (GO:0001934 and GO:0006464), intracellular signal transduction (GO:1902533), and DNA transcription (GO:0045893). The viruses also developed immune evasion (Kasuga et al., 2021) to suppress cytokine-mediated signaling (GO:0019221) and host defense response (GO:0031349). Gene products located at endosomes and lysosomes (GO:0010008 and GO:0005764) were also down-regulated, probably because the viruses hijacked these organelles for their own manufacturing. Meanwhile, the host protein biosynthesis machinery was highly activated for the viral replication, such as macromolecule biosynthesis (GO:0034645), cotranslational protein targeting to the membrane (GO:0006613), peptide biosynthesis (GO:0043043), large ribosomal subunit (GO:0015934), and mitochondria (GO:0005759) (Gatti et al., 2020; V’kovski et al., 2021). In line with our previous study (Xing et al., 2021), the microtubule cytoskeleton system was utilized for viral transportation. Of interest, cell cycle pathways were also up-regulated, including DNA replication and repair (GO:0006260 and GO: 0006281), mitotic cell cycle transition (GO:1901990), and P53 signaling (GO:1901796).

For the second task, 49 RNA-seq datasets of SARS-CoV-2 infection were incorporated to generate valid SARS-CoV-2 signatures, yielding a set of 23 valid SARS/MERS/SARS-CoV-2 infection signatures in total (i.e., robust CoV signatures v2, Figure 3C, Data S7). In an external positive drug set (Data S1), the reversal of CoV signatures v2 could predict known CoV inhibitors (AU-ROC = 0.76) and significantly correlated with drug EC_50_ values (Spearman R = 0.47, p = 0.005, Figure 3C). Most of the invalid CoV signatures showed no enrichment of positive drugs (grey curves in Figures 3A and 3C), suggesting their irrelevance with anti-CoV drug discovery. Of interest, several invalid CoV signatures enriched the positive drugs at the right end, which indicates the mimicking of host antiviral response by those host-targeting drugs. Similar patterns were observed in the published CoV signatures as well.

The valid CoV signatures captured essential biology of how CoVs hijack the host cell machinery. Through GO enrichment analysis on the updated CoV signatures (Data S6), we observed that general pathway enrichment patterns remained similar to CoV signatures v1, i.e., inhibiting host activity and antiviral response (Kasuga et al., 2021; Lei et al., 2020; Schroeder et al., 2021; Xia et al., 2020) while boosting protein and RNA biosynthesis (Figure 3D). More specific than v1, the CoV signatures v2 highlighted down-regulation of cell migration (GO:0030335) and neutrophil-mediated immunity (GO:0002446). Another minor difference is that virus hijacked ATP synthesis on mitochondria inner membrane (GO:0042775) was more significant in v2 signatures, whereas v1 enriched genes were expressed in the mitochondrial matrix. The valid CoV signatures (v2) maintain some diversity in biological pathways and form complementary clusters (Figure S4A). Compared with the above mentioned published COVID-19 signatures, the selected valid CoV signatures are richer in pathway presentation (Figure S4). Unlike the published signatures, which mainly focus on interferon pathway over-activation, the valid signatures proposed in this work captured the immune evasive nature of these CoVs at the early stage of infection (Figures S4C and S4D), which is essential in regulating host antiviral processes to fight against different variants.

We also sought to answer the question of why, among hundreds of infection versus control comparisons, only 23 were informative for drug screening. First, CoV infection dysregulated pathways showed diverse and complicated dynamic patterns over different time points and varied across different research models (Figure S5A), meaning not all comparisons are valid for drug screening. Second, by examining the SARS-CoV-2-induced transcriptomic changes in different tissues, we found that infection profiles of lung and PBMC followed similar patterns with the CoV meta-signature v2 (Spearman Rho: 0.62 and 0.48), whereas no obvious pattern was observed in liver, kidney, and heart samples (Figure S5B). Because the lung is the primary target organ for COVID-19, and PBMCs circulating in the blood are highly exposed to SARS-CoV-2, the transcriptomic changes in these two organs are considered appropriate to characterize the viral infection. These observations emphasize the power of data-driven approaches in teasing out useful signals for therapeutic discovery.

### The CoV signature genes separate COVID-19 patients from controls

**Figure 4:**
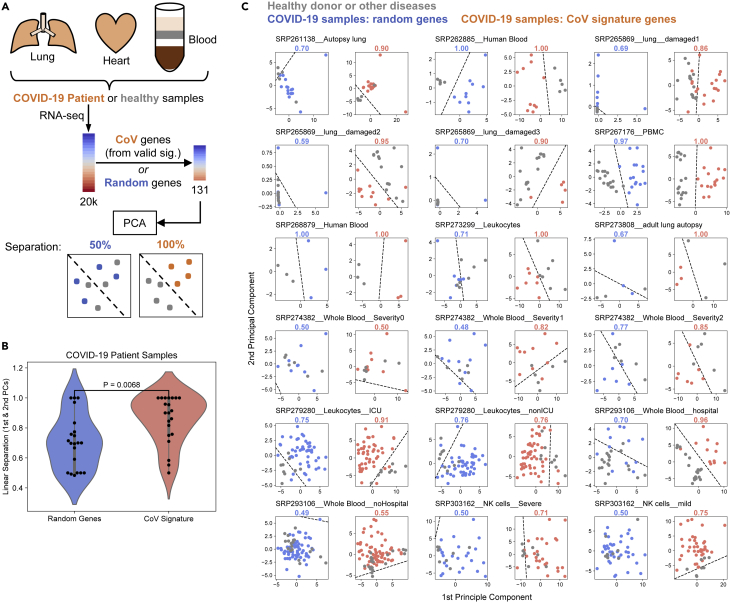
Verifying the CoV meta-signature v1 (derived from SARS- or MERS-infected host transcriptomic profiles) with COVID-19 patient cohorts (A) Workflow of patient sample analysis for the CoV meta-signature validation. (B) Violin plot to compare how well COVID-19 patient samples are separated from others using the CoV signature genes or random genes. Each point indicates the classification accuracy for a patient group. The p-value was computed from the Wilcoxon rank-sum test. (C) Examples of PCA plots of RNA-seq samples from different COVID-19 patient groups. Cohort IDs, tissues, and severities are denoted in group titles. For each comparison, the left PCA plot was derived from a random gene expression matrix of COVID-19 patient (blue) or healthy/other disease (grey) samples, whereas the right PCA plot was based on the CoV signature genes where orange scatters indicate COVID-19 patient samples. For each PCA plot, a dashed line indicates the boundary between COVID-19 samples and others (derived from linear discriminant analysis, see STAR Methods), and the separation accuracy is also labeled above the plot. PCA: principal component analysis. See also Figure S6.

To confirm that the selected disease signatures capture essential COVID-19 pathological processes, we first created a summarized CoV meta-signature based on the 13 valid signatures (v1), with 88 genes having a positive effect size and 43 genes having a negative effect size. Furthermore, we collected 14 studies comprising 21 patient cohorts (Data S8) with 560 whole-transcriptomic samples from different organs (e.g., lung, heart, and blood) of COVID-19 patients, healthy donors, and patients who had died of non-infectious diseases. For each cohort, principal component analysis (PCA) was performed using the 131 CoV signature genes or 131 random genes to visualize the separation of COVID-19 patient samples from others (Figure 4A). As expected, the CoV signature genes significantly outperformed random genes in separating COVID-19 samples from those of healthy donors or other diseases (Figure 4B, within the 2D space defined by the first two principal components). For example, in a study of lung autopsies of deceased patients, CoV signature genes correctly classified 90% of the COVID-19 patients, whereas the random genes only showed a 70% accuracy (Figure 4C, dataset SRP261138). Note that when two groups of samples present distinct transcriptomic profiles, even the random genes could separate them although the set of CoV signature genes performs better. The CoV signature genes were also found to associate with symptom severity. Using data from a study of lung autopsy (SRP265869), we observed 95% and 90% of classification accuracy were achieved by the selected genes for degree 2 and 3 lung damaged patients, yet the separation was reduced to 86% in degree 1 (Figure 4C). Using the data from a whole-blood study (SRP274382), the separation accuracy increased as severity elevated (the accuracies in severity degree of 0, 1, and 2 were 50%, 82%, and 85%, respectively) (Figure 4C). Similarly, in studies SRP279280 and SRP293106, the CoV signature performed better in intensive care unit (ICU) patients than in non-ICU patients and better in hospitalized patients than in non-hospitalized patients (Figure 4C). Similarly, we evaluated the CoV meta-signature using data from SARS-CoV-2 infected preclinical models (cell lines and organoids) and found the separation was also significant (Figure S6). These results suggested that the proposed CoV meta-signature successfully characterized the critical features of SARS-CoV-2 infection.

### The CoV signatures led to the discovery of IMD-0354 as a potent candidate against SARS-CoV-2 and its variants

**Figure 5:**
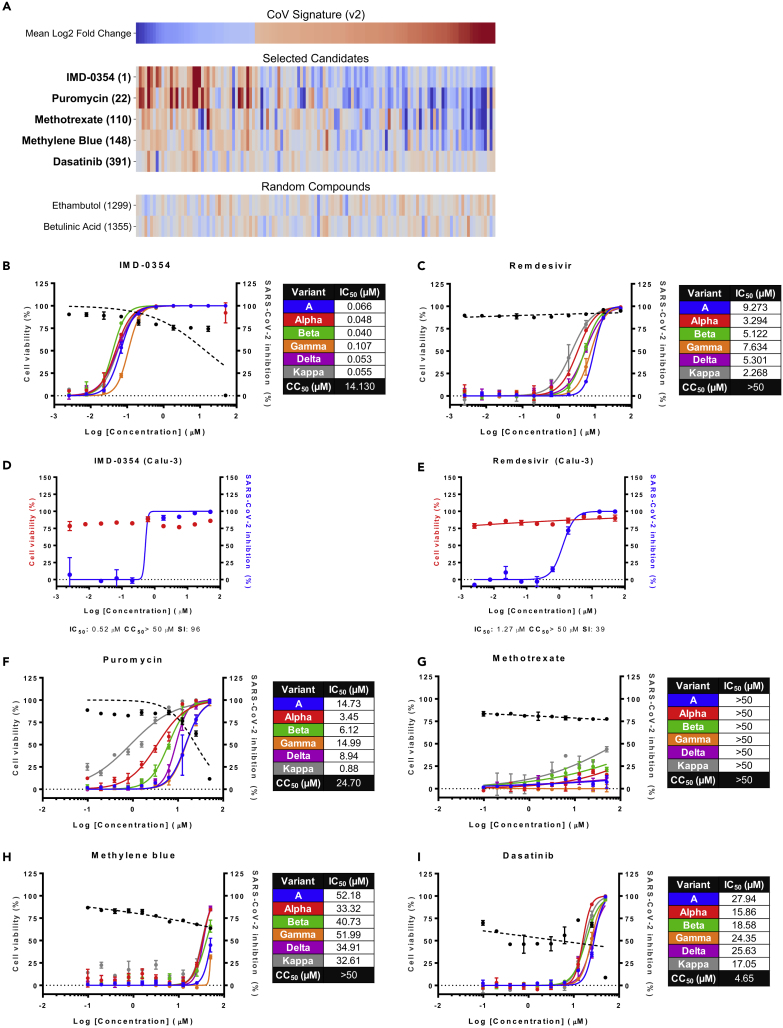
Selected candidates based on the reversal of valid CoV signatures and their *in vitro* efficacy against SARS-CoV-2 and its variants (A) Transcriptomic profiles of five selected and two random compounds aligned to the CoV meta-signature v2. Red and blue indicate up-regulation and down-regulation, respectively. The rank of each compound was annotated after the drug name. (B, C, and F–I) Dose-response curves of compounds inhibiting SARS-CoV-2 and variants replication and their cytotoxicity in Vero cells. IC_50_ and CC_50_ values are labeled on the right side. D and E, Dose-response curves of IMD-0354 and remdesivir inhibiting SARS-CoV-2 replication and their cytotoxicity in Calu-3 cells. B–I, IC_50_ and CC_50_ values were measured in duplicates.

Next, we applied the valid CoV signatures v2 to drug prediction for SARS-CoV-2 variants. The candidate ranking list was also updated (Data S9), with IMD-0354 (IKK-2-inhibitor-V) at the top. Its transcriptomic profiles presented a clear reversal pattern compared with CoV meta-signature v2 (Figure 5A). In line with the prediction, IMD-0354 demonstrated potent inhibitory activity against six variant viruses in VeroE6 cells, with IC_50_ values around 50 nM for the lineage A virus (ancestral SARS-CoV-2), Alpha, Beta, Delta, and Kappa variants, and 107 nM for the Gamma variant. With a CC_50_ of 14 μM, IMD-0354 showed a wide therapeutic window of 132-folds (Figure 5B). Kato et al. (Kato et al., 2020, p. 0354) also reported its prevention of the ancestral SARS-CoV-2-induced CPE on VeroE6/TMPRSS2 cells, confirming our results. Strikingly, IMD-0354 is 90-fold more active than remdesivir (IC_50_ 2–10 μM, Figure 5C) to the six variants tested. In Calu-3 cells, the antiviral IC_50_ of IMD-0354 was 0.52 μM with no observable cytotoxicity (Figure 5D), whereas the IC_50_ of remdesivir was 1.27 μM (Figure 5E). IMD-0354 is structurally similar to niclosamide, an anthelmintic that elicits broad-spectrum antiviral activity (Xu et al., 2020) and is under a Phase II clinical trial for COVID-19 (NCT04399356). More importantly, IMD-1041, the prodrug of IMD-0354, has completed a Phase I clinical trial and is under Phase II investigation for chronic obstructive pulmonary disease (COPD) (NCT00883584) and pulmonary fibrosis (IMMD Drug Discovery Business), indicating its high safety and reasonable bioavailability for lung diseases.

We also tested the cellular antiviral activity of four other compounds showing varying predicted priorities, i.e., puromycin (ranked 22), methotrexate (ranked 110), methylene blue (ranked 148), and dasatinib (ranked 391) (Figure 5A). Consistent with the ranking, puromycin inhibited Kappa viral replication with an IC_50_ of 0.88 μM and inhibited other variants with IC_50_ values ranging from 3 to 15 μM, suggesting a moderate activity (Figure 5F). Methotrexate and methylene blue were active only at the highest concentration tested (Figures 5G and 5H). Dasatinib, the least prioritized one, although showed moderate activity (IC_50_ ∼ 20μM), its CC_50_ was even lower (4.65 μM), suggesting a nonspecific inhibition to the host cell activities instead of specifically targeting the CoV signatures (Figure 5I). These *in vitro* validation results confirmed the robustness of our CoV signatures and the rationale of drug screening based on the reversal of infected host gene expression. Together, we propose IMD-0354 or its prodrug as a drug candidate for SARS-CoV-2 and its variants.

### IMD-0354 inhibits viral infection through type I interferon stimulation and other polypharmacological mechanisms

**Figure 6:**
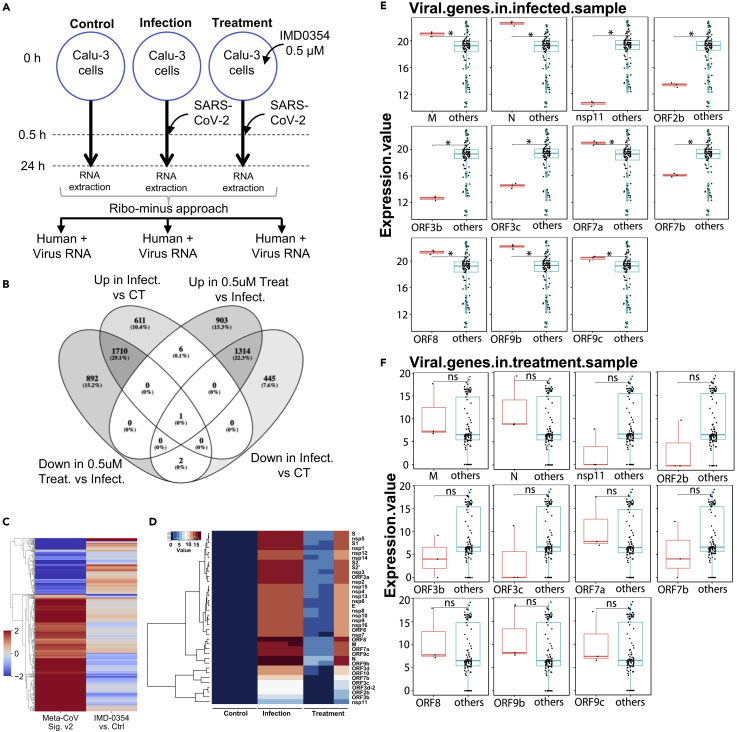
Host gene expression response upon the treatment of IMD-0354 in SARS-CoV-2 infected cells (A) Workflow of RNA-seq library preparation to capture human and viral RNAs. Each group contains three replicates. (B) Venn diagram showing the comparison of differentially expressed (DE) genes obtained between Infection versus Control (CT) and 0.5 μM Treatment versus Infection. (C) A cluster-map showing the reversal of DE genes in the meta-CoV signature (v2) as compared to the treatment group (IMD-0354 0.5 μM versus Control). (D) Expression values of all SARS-CoV-2 viral proteins in Control, Infection, and 0.5 μM treatment samples. (E) Viral proteins showing variations (Wilcoxon rank-sum test, p-value < 0.05) with respect to total proteins in infected cells. (F) The expression of viral proteins is neutralized in the 0.5 μM IMD-0354 treatment group. ns: not significant (Wilcoxon rank-sum test). In (C and D), the blue color represents down-regulated genes, and the red color represents up-regulated genes. In (E and F), box top- and bottom-edges indicate 25 and 75 percentiles, and the horizontal line inside the box indicates the median.

To gain better insights into the mechanism of action (MoA) of IMD-0354 on viral inhibitory processes, we performed RNA-seq based on three sets of experiments, including control, SARS-CoV-2 infection, and IMD-0354 treatment followed by SARS-CoV-2 infection in Calu-3 cells (Figure 6A). We used the ribo-minus approach for RNA-seq library preparation to capture the host mRNA as well as viral RNA. After mapping the sequencing reads to the human transcriptome, we obtained 4089 differentially expressed (DE) genes in the infection group compared to the control, and 4828 DE genes in the treatment compared to the infection group (Figure 6B and Data S10). The gene expression clustering presents a counteracting pattern between the summarized meta-CoV signature and the IMD-0354 treated group (Figure 6C), confirming our initial hypothesis that IMD-0354 could reverse the COVID-19 infection-induced gene expression pattern. We also mapped the sequencing reads to the SARS-CoV-2 genome and observed minimal/no expression of viral genes in control and treatment samples, yet high expression of viral genes in infected samples (Figure 6D). In detail, the viral M, N, ORF7a, ORF8, ORF9b, and ORF9c showed higher expression (read counts normalized with gene length) than other genes (Wilcox rank-sum test, p-value < 0.05, two-sided; Figure 6E); whereas NSP11, ORF2b, ORF3b, ORF3c, and ORF7b were least expressed compared with other genes. Of interest, in IMD-0354-treated samples, expression of all viral genes was at the same and low level, suggesting complete viral inhibition (Figure 6F).

**Figure 7:**
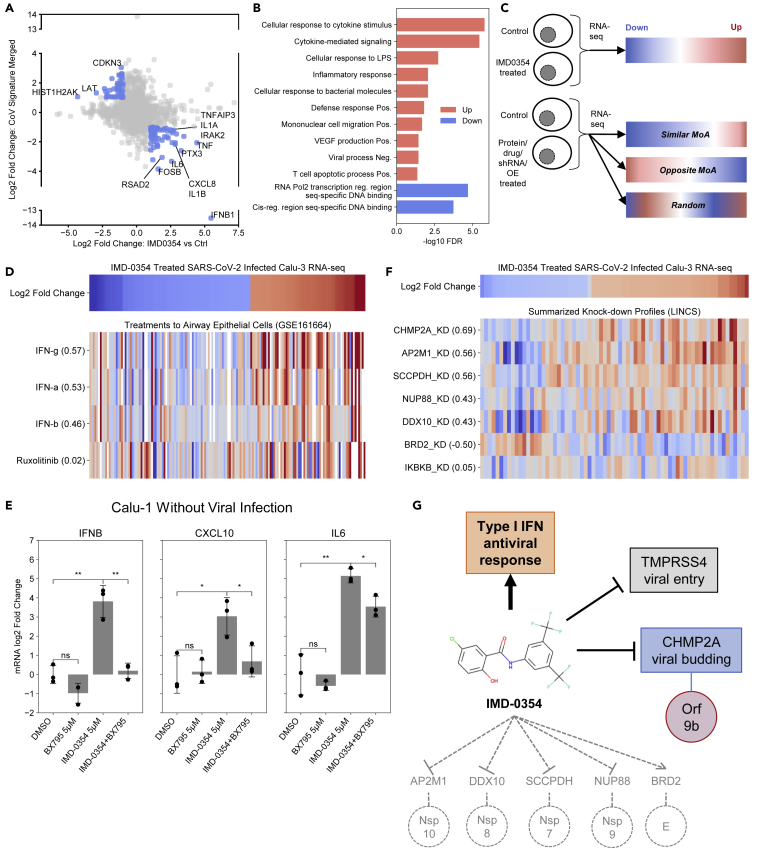
IMD-0354 induces type I interferon, blocks viral entry, and inhibits several host proteins hijacked by the virus (A) Scattering plot highlighting CoV signature genes reversed by IMD-0354. The X axis indicates the log_2_ fold changes between the treatment group and the control group, as illustrated in Figure 6A. The Y axis indicates averaged log_2_ fold changes of CoV valid signatures (v2). Blue points denote genes dysregulated by CoV infection and significantly reversed by IMD-0354 treatment. (B) Enriched up- (red) and down- (blue) regulated pathways in IMD-0354 induced host gene expression change (treatment group versus control group as in Figure 6A). The X axis indicates p-values (FDR corrected and -log10 transformed) computed from Fisher exact test. (C) Illustration of the MoA query approach based on transcriptomic data. OE: over-expression. (D) Transcriptomic changes induced by IMD-0354, interferons, and ruxolitinib. Whole transcriptomic data of IMD-0354 were generated as shown in Figure 6A, and other treatment profiles were downloaded from GEO (GSE161664). Values in brackets denote RGES of a treatment profile compared with the IMD-0354 profile. IFN: interferon. (E) RT-qPCR results on the expression change (log_2_ transformed) of *IFN-β,CXCL10*, and *IL6* by different compound treatments compared with DMSO in Calu-1 cells without viral infection. Each dot represents a replicate, each bar represents the average of three replicates. Error bars denote standard deviations (n = 3). ∗: p-value < 0.05; ∗∗: p-value < 0.01, ns: not significant; Student’s *t* test. (F) Transcriptomic changes induced by IMD-0354 and shRNA perturbagens. Settings are the same as D except that shRNA profiles were from the LINCS L1000 dataset (landmark genes only). In B, C, D, and F, red indicates up-regulation, blue indicates down-regulation and white indicates missing values. (G) Proposed antiviral mechanism of IMD-0354, including type I interferon response induction, viral entry blockade via *TMPRSS4* inhibition, and viral budding blockade through *CHMP2A* down-regulation, together with regulating host proteins hijacked by the virus. Main antiviral mechanisms are highlighted with colors. Circles indicate SARS-CoV-2 proteins directly interacting with host proteins. See also Table S5, Figures S7 and S8.

We further investigated these RNA-seq samples to delineate the MoA. With a minimal amount of viral RNA in the treatment group, the RNA-seq profiles mainly captured the effect of IMD-0354 pre-treatment on host cells. Compared with the merged CoV signature (v2, mean log_2_ fold change), several innate immune response genes inhibited by the virus were significantly up-regulated in the IMD-0354 treated group, including *IFNB1*, *IL1B*, *IL6*, and *TNF* (Figure 7A and Data S11). GO enrichment analysis also suggested up-regulation of cytokine-mediated signaling, viral process negative regulation, and NF-kB transcription factor activation (Figure 7B and Data S12). These results suggest that IMD-0354 blocks viral replication by boosting the antiviral response in lung epithelial cells. Based on the assumption that perturbagens with similar transcriptomic profiles share similar MoA (Figure 7C), we used the IMD-0354 RNA-seq profile to query gene knock-down and over-expression profiles in LINCS. The transcriptomic changes induced by over-expression of *IFNG*, *TIRAP*, or *IFNB1* mimicked IMD-0354 with high RGES values of 0.83, 0.63, and 0.58, respectively (Data S13). Similarly, the whole-transcriptomic profiles of interferons on small airway epithelial cells also exhibited similar patterns with IMD-0354 (RGES ∼0.5); whereas ruxolitinib, a JAK inhibitor, showed no correlation (RGES = 0.02, Figure 7D and Data S10, data source: GSE161664). By incubating Calu-1 cells with IMD-0354 (without subsequent viral infection), we also observed increased expression of *IFN-β*, *CXCL10*, and *IL-6* with 15, 9, and 36-folds, respectively; and the activation of these genes was counteracted by BX795, a TBK1/IKKε inhibitor that blocks *IFN-β* production (Figure 7E and Table S5). These results suggest that IMD-0354 pre-treatment activates interferon-mediated antiviral signaling, which has been proven to inhibit SARS-CoV-2 infection (Li et al., 2021). IMD-0354 did not over-activate *ACE2*, a receptor of SARS-CoV-2 and also an interferon-stimulated gene (log_2_ fold change 0.47, Data S10). IMD-0354 did not stimulate *IFN-β* or *IL-6* in macrophages, meaning this drug might not worsen the cytokine storm caused by COVID-19 (Figure S7). Although BX795 and ruxolitinib inhibit type-I interferon response, neither of them abrogated the antiviral effect of IMD-0354 (Figure S8), suggesting other potential pathways regulated by IMD-0354. In addition, the knock-down of *CHMP2A*, a core component of the endosomal sorting required for transport complex III involved in virus budding (Votteler and Sundquist, 2013), also resembled the IMD-0354 RNA-seq profile (RGES = 0.69, Figure 7F and Data S14). *CHMP2A* directly interacts with ORF9b of SARS-CoV-2 (Gordon et al., 2020), which was found hyperactively expressed during infection but suppressed by IMD-0354 treatment (Figures 6E and 6F). Thus, we reason that IMD-0354 might down-regulate *CHMP2A* expression to block SARS-CoV-2 release to neighbor cells. Also, IMD-0354 might restore SARS-CoV-2-hijacked genes (Gordon et al., 2020) by up-regulating *BRD2* and down-regulating *AP2M1*, *SCCPDH*, *NUP88*, and *DDX10* (Figure 7F and Data S14). Of interest, *IKBKB* inhibition, the original MoA of IMD-0354, might not contribute to its antiviral effect (Figure 7F). Moreover, IMD-0354 inhibits *TMPRSS4* (Kang et al., 2013), one of the crucial enzymes for SARS-CoV-2 entry (Zang et al., 2020), which could partially explain its higher EC_50_ in Calu-3 cells than VeroE6 (Figures 5B and 5D), as *TMPRSS4* is elevated in Calu-3. Taken together, we propose that IMD-0354 inhibits SARS-CoV-2 replication mainly through inducing type I interferon-mediated antiviral response, together with multiple polypharmacological mechanisms including *TMPRSS4* inhibition mediated viral entry blockade, *CHMP2A* down-regulation mediated virus budding inhibition, and regulation of host proteins hijacked by the virus (Figure 7G).

## Discussion

SARS-CoV-2 induces variable responses in humans, causing highly diverse pathological symptoms. Thus, a therapeutic agent that targets only a single pathway or gene may only lead to weak inhibition. System-based approaches hold great promise to complement the traditional target-based approaches and have been employed to propose drug repurposing candidates for COVID-19 (Bocci et al., 2020; Le et al., 2021; Zhou et al., 2020). One of the system-based strategies is to propose drugs to reverse disease gene expression (Qu and Rajpal, 2012), which has demonstrated successes in drug repurposing in various diseases (Chen et al., 2021a; Dhindsa et al., 2021; Mun et al., 2020; Qu and Rajpal, 2012). More than 1,000 RNA-seq profiles of SARS-CoV-2 infected patients and preclinical models are publicly available, forming a rich resource of COVID-19 signatures for repurposing drugs. However, the pathological relevance of the CoV infection signature is critical for antiviral drug discovery. For example, those COVID-19 signatures focusing on over-activated host immune response do not present a clear pathological effect of virus hijacking the host cell machinery or immune escaping. We found the raw CoV signatures displayed diverse patterns of enriched pathways, and only 6% of them could recover published anti-SARS-CoV-2 active compounds, which motivated us to develop a computational system-based framework to select disease signatures relevant to therapeutic discovery against SARS-CoV-2 and its current and emerging variants.

Inspired by the high correlation of *in vitro* drug efficacy data between SARS-CoV and MERS-CoV, we distilled knowledge from published inhibitors of related coronaviruses to evaluate the pathological relevance of the CoV signatures and applied the valid signatures to drug discovery against new variants. Because SARS-CoV, MERS-CoV and SARS-CoV-2 have highly similar genomes (Lu et al., 2020), it is likely that they elicit similar dysregulations of cellular machinery that affect host genes and pathways. The positive enrichment of the SARS-CoV-2 inhibitors in the hits predicted from the valid SARS and MERS signatures further supported that drug candidates identified with this method would be active against new variants. Indeed, the promising *in vitro* results of the top candidate IMD-0354 in the inhibition of SARS-CoV-2 and six VOC supported our hypothesis. Thus, by using legacy profiles from the same virus family and compound efficacy against previous viruses, we can quickly narrow down the candidate list for emerging viruses whose profiles are often not readily available.

Among hundreds of possible signatures, only a small fraction of them are informative in drug prediction. Our data-driven approach elegantly teased out those informative signatures, resulting in a robust meta-signature of CoV-hijacking host transcriptomic change. Although this signature was initially derived from SARS and MERS data, it retains the power to reclassify the SARS-CoV-2 infected and control patient samples in multiple independent datasets. Furthermore, the valid SARS-CoV-2 signatures were merged with SARS-CoV and MERS-CoV signatures to create a refined CoV signature. Pathway analysis of these valid signatures revealed the dysregulation of several known viral pathways. For example, the antiviral immune response was down-regulated in our signatures and was in concordance with previous reports (Kasuga et al., 2021; Xia et al., 2020). In addition, replication, mitochondrial ATP synthesis, translation, and peptide synthesis-related pathways required for viral multiplication were up-regulated in these signatures (Chen et al., 2021b; deBreyne et al., 2020; Gatti et al., 2020; V’kovski et al., 2021). A drug candidate exhibiting strong antiviral effects against SARS-CoV-2 is expected to target one or multiple dysregulated pathways.

The RNA-seq analysis of our drug-treated samples and SARS-CoV-2 infected samples showed the inverse relationship on different pathways, in agreement with our hypothesis that IMD-0354 targets multiple pathways dysregulated by the virus. In addition, the viral gene expression analysis depicted the hyperactivation of SARS-CoV-2 proteins, including M, N, ORF7a, ORF8, ORF9b, and ORF9c in infected samples. The M protein and ORF7a are known for assembling and budding viral particles and are involved in recruiting structural proteins to ER-Golgi intermediate compartments (Nelson et al., 2005; Voss et al., 2009). The N protein is involved in genome protection, viral RNA replication, virion assembly, and immune evasion (including IFN-I suppression) (Cubuk et al., 2021; Mu et al., 2020, p. 1; Wang et al., 2021). The ORF8 protein is crucial in viral assembly and immune invasion *via* inhibiting type I interferon signaling (Li et al., 2020, p. 6; Zhang et al., 2021, p. 6). The ORF9b protein dysregulates mitochondrial function (Shi et al., 2014; Wu et al., 2021), and the ORF9c protein interacts with various host proteins including Sigma receptors, implying involvement in lipid remodeling and the ER stress response (Gordon et al., 2020). The absence of these genes in IMD-0354 treated cells suggests a complete viral infection inhibition under 0.5 μM.

The comparison of the merged CoV signature and IMD-0354-induced transcriptomic profiles revealed that IMD-0354 activated interferon pathway-related genes (e.g., *IFNB1*, *IL1B*, *IL6*, and *CXCL8*). Several studies suggested that type I interferon activation leads to the blockage of SARS-CoV-2 infection (Kim and Shin, 2021; Lei et al., 2020; Pierce Carl et al., 2020; Schroeder et al., 2021; van der Wijst Monique et al., 2021; Xia et al., 2020). Independent validation by qPCR further confirmed that IMD-0354 stimulated the interferon pathway even without any viral challenge. This was also supported by the analysis of viral proteins, where we observed activation of viral N protein, an interferon inhibitor, in infected samples but down-regulation of viral N protein in the IMD-0354 treated samples. In addition, down-regulation of *CHMP2A* could lead to viral inhibition as it is a member of the endosomal sorting complex required for transport (ESCRT), a cellular machinery hijacked by the virus for its replication and release (Calistri et al., 2021). *TMPRSS4* and a similar serine protease *TMPRSS2* mediate SARS-CoV-2 entry into cells with an additive effect by inducing cleavage of the S protein and enhancing membrane fusion (Wruck and Adjaye, 2020; Zang et al., 2020), and *TMPRSS2* blockage resulted in SARS-CoV-2 inhibition (Hoffmann et al., 2020). In addition, the knock-down profile of several host proteins hijacked by the virus such as AP2M1 and NUP88 also showed significant correlations with the IMD-0354-induced profile. Collectively, the inhibition of these proteins by IMD-0354 would effectively inhibit viral replication, as evidenced by the negligible viral gene expression in the RNA-seq results of drug-treated samples, which resembled uninfected control samples (Figure 6D). Together, these suggest the efficiency of our system-based host-transcriptome-targeting approach in the discovery of novel antiviral drugs with polypharmacological effects. Our robust CoV signatures would be instrumental in the future also to discover novel anti-CoV drugs even with very limited transcriptomic profiles.

In conclusion, by incorporating the knowledge of published CoV inhibitors and aggregating the disease signatures of SARS-CoV, MERS-CoV, and SARS-CoV-2 in a data-driven fashion, we provide a collection of robust CoV signatures to enable drug discovery for new variants, which led us to discover a potent drug candidate, IMD-0354, with anti-SARS-CoV-2 activity, which also showed enhanced potency against several variants. Our MoA analysis suggests that IMD-0354 activates type I interferon antiviral response and targets multiple pathways involved in the viral life cycle, suggesting the power of this system-based approach for drug discovery and the importance of robust pathological relevant CoV signatures. This pipeline might provide more robust CoV signatures as a forward-looking tool to enable drug discovery for new variants and future potential pandemics by iteratively updating CoV infection profiles and anti-CoV drug profiles.

### Limitations of the study

Here are some limitations of our work and how they can be overcome. Because we used the transcriptome profiles from different *in vivo* and *in vitro* models, which might add the biases and limitations in their disease signatures, to overcome the limitation of technical variations, we created the disease signatures of all datasets with one pipeline and made comparisons within individual studies. In addition, diverse *in vivo* and *in vitro* models were included to capture all possible transcriptional dysregulated profiles and only those that could inform drug discovery were selected to create a robust disease signature for SARS-CoV-2. Although our pipeline offers a unique approach to discovering potent drug candidates for future pandemics, it requires prior knowledge of transcriptomics profiles and active compound profiles of any of the family members related to the infection particle. This limitation could be overcome with the advancement and wide availability of sequencing technologies. The drug reversal analysis used the drug profiles from the LINCS, which are based on cancer cells and only cover 978 landmark genes. Although this database has already been successfully applied for drug predictions in various non-oncology diseases, a drug profile library database specific to viral perturbation is expected to improve performance. Lastly, the IMD-0354 prodrug IMD-1041 is even more promising because it is orally available and has been investigated for COPD, a group of lung diseases that block airflow and make it difficult to breathe. However, because this drug is currently owned by a private company and relevant information, including chemical structure, is not available, it is not feasible to immediately launch animal studies to fully evaluate its candidacy.

## STAR★Methods

### Key resources table

**Table:** 

REAGENT or RESOURCE	SOURCE	IDENTIFIER
**Antibodies**		

Anti-SARS-CoV-2 N protein	Sino Biological Inc. (Beijing, China)	
Alexa Fluor 488 goat anti-rabbit IgG (H + L) secondary antibody	Molecular Probes (Eugene, OR)	Cat# A-11008, RRID:AB_143165

**Bacterial and virus strains**		

βCoV/KOR/KCDC03/2020 (ancestral SARS-CoV-2)	Korea Disease Control and Prevention Agency (KDCA)	
hCoV-19/Korea/KDCA51463/2021 (alpha)	Korea Disease Control and Prevention Agency (KDCA)	
hCoV-19/Korea/KDCA55905/2021 (beta)	Korea Disease Control and Prevention Agency (KDCA)	
hCoV-19/Korea/KDCA95637/2021 (gamma)	Korea Disease Control and Prevention Agency (KDCA)	
hCoV-19/Korea/KDCA119861/2021 (delta)	Korea Disease Control and Prevention Agency (KDCA)	
hCoV-19/Korea/KDCA105288/2021 (kappa)	Korea Disease Control and Prevention Agency (KDCA)	

**Chemicals, peptides, and recombinant proteins**		

IMD-0354	MedChemExpress (Monmouth Junction, NJ)	HY-10172
Puromycin	MedChemExpress (Monmouth Junction, NJ)	HY-B1743A
Methotrexate	MedChemExpress (Monmouth Junction, NJ)	HY-14519
Methylene blue	MedChemExpress (Monmouth Junction, NJ)	HY-14536
Dasatinib	MedChemExpress (Monmouth Junction, NJ)	HY-10181
Bortezomib	Selleckchem (Houston, TX)	S1013
Tyloxapol	Selleckchem (Houston, TX)	S4578
Nisoldipine	Selleckchem (Houston, TX)	S1748
Nvp-bez235	Selleckchem (Houston, TX)	S1009
Fluvastatin	Selleckchem (Houston, TX)	S1909
Alvocidib	Selleckchem (Houston, TX)	S1230
Chloroquine	Cayman Chemical (Ann Arbor, MI)	14194
Remdesivir	MedChemExpress (Monmouth Junction, NJ)	HY-104077
Ruxolitinib	MedChemExpress (Monmouth Junction, NJ)	HY-50856
diABZI	MedChemExpress (Monmouth Junction, NJ)	HY-123943
BX795	MedChemExpress (Monmouth Junction, NJ)	HY-10514

**Critical commercial assays**		

MagMAX^TM^ mirVana^TM^ Total RNA Isolation Kit	ThermoFisher	A27828
KAPA RNA HyperPrep Kit	Roche	
RNA extraction reagent	Vazyme, Nanjing, China	R401-01
HiScript II Q RT SuperMix	Vazyme, Nanjing, China	R223-01
ChamQ SYBR qPCR Master Mix	Vazyme, Nanjing, China	Q331-02
HiScript III RT SuperMix	Vazyme, Nanjing, China	R323-01

**Deposited data**		

RNA-seq data for control, SARS-CoV-2 infected, and IMD-0354 treated Calu-3 samples	This paper	GEO: GSE187420

**Experimental models: Cell lines**		

African green monkey: Vero cells	AmericanType Culture Collection (ATCC, Manassas, VA, USA)	CCL-81; CRL-1586
Human: Calu-3 cells	AmericanType Culture Collection (ATCC, Manassas, VA, USA)	HTB-55
Human: Calu-1 cells	AmericanType Culture Collection (ATCC, Manassas, VA, USA)	HTB-54
Human: THP-1 cells	AmericanType Culture Collection (ATCC, Manassas, VA, USA)	TIB-202

**Oligonucleotides**		

Primers for human ACTB: Forward: catgtacgttgctatccaggc Reverse: ctccttaatgtcacgcacgat	This paper	
Primers for human IFNB: Forward: cagcatctgctggttgaaga Reverse: cattacctgaaggccaagga	This paper	
Primers for human CXCL10: Forward: ccacgtgttgagatcattgct Reverse: tgcatcgattttgctcccct	This paper	
Primers for human IL6: Forward: ttcggtccagttgccttctc Reverse: tacatgtctcctttctcagggc	This paper	

**Software and algorithms**		

R (3.5.1)	https://www.r-project.org/	
Python (3.7)	https://www.python.org/	
ggplot2	https://ggplot2.tidyverse.org/	
Pheatmap	https://www.rdocumentation.org/packages/pheatmap/versions/1.0.12	
Matplotlib	https://matplotlib.org/	
Seaborn	https://seaborn.pydata.org/	
Sci-kit learn	https://scikit-learn.org/stable/index.html	
STAR	Dobin et al., 2013	https://github.com/alexdobin/STAR
edgeR	Robinson et al., 2010	https://bioconductor.org/packages/release/bioc/html/edgeR.html
Prisim 7 (GraphPad)	San Diego, CA	
Columbus	Perkin Elmer,Waltham, MA	
OCTAD	Zeng et al., 2021	http://octad.org/

### Resource availability

#### Lead contact

Further information and requests for resources and reagents should be directed to and will be fulfilled by the lead contact, Bin Chen (chenbi12@msu.edu).

#### Materials availability

This study did not generate new unique reagents.

### Experimental model and subject details

#### Cell culture

Vero cells were maintained at 37°C with 5% CO_2_ in Dulbecco’s Modified Eagle’s Medium, supplemented with 10% heat-inactivated fetal bovine serum (FBS) and 1X Antibiotic-Antimycotic solution. Calu-3 used in this study is a clonal isolate, which shows a higher growth rate compared to the parental Calu-3 obtained from the AmericanType Culture Collection (ATCC HTB-55). Calu-3 was maintained at 37°C with 5% CO_2_ in Eagle’s Minimum Essential Medium, supplemented with 20% heat-inactivated fetal bovine serum (FBS), 1X MEM-NEAA and 1X Antibiotic-Antimycotic solution. Calu-1 cells were cultured in McCOY’s 5A medium supplemented with 10% Fetal Bovine Serum (FBS) and 2.2 g/L NaHCO_3_. Calu-1 cells were incubated at 37°C under 5% (v/v) CO_2_ atmosphere. For the pilot screening, Vero E6 cells [CRL:1586, ATCC] were grown in Eagle’s minimal essential medium (EMEM) supplemented with penicillin (100 units/mL), streptomycin (100 μg/mL), and 10% fetal bovine serum (FBS).

### Method details

#### Experimental design

This study aims to discover drug repurposing candidates for COVID-19 and emerging VOC using a computational system-based approach, which ranks drugs based on their predicted potency to reverse the CoV host response signature (i.e., host gene expression dysregulated in COVID-19 samples). We made tremendous efforts to decipher the complexity and variance of host responses through large-scale analysis of published data. When collecting and processing the CoV transcriptomic profiles, we used the same pipeline to process all the raw sequence data and made the comparison within the same study to minimize the batch effect. Sample annotation followed the original study, and no samples were purposely excluded from the analysis. Published anti-CoV compounds (i.e., positive controls) were divided into a calibration set and a testing set to select and validate CoV signatures for drug discovery. We screened the LINCS drug-treated transcriptomic profiles to prioritize repurposed candidates that can reverse the robust CoV infection signatures. The experimental validation of the repurposed drug candidates for inhibiting SARS-CoV-2 and its VOC was performed on two frequently used cell lines, namely Calu-3 and Vero-E6. The MoA of the most potent drug was also explored based on RNA-seq profiling and perturbagen connectivity scoring, followed by independent q-PCR confirmation. Technical and biological replicates in each experiment were used to check the robustness of the results.

#### MSigDB hallmark gene enrichment **analysis**

Fisher exact tests were performed to calculate the p value of a gene set enrichment in the up- or down-regulated genes from a published COVID-19 signature or a raw CoV signature. The background was defined as a union of all genes annotated in MSigDB Hallmark dataset. For improved visualization, the p values were transformed by -log10 for up-regulation enrichment and by +log10 for down-regulation enrichment, and we took the one with a larger absolute value as the final direction.

#### Computation of infection signatures

SARS-CoV, MERS-CoV, and SARS-CoV-2 related data were retrieved from ArrayExpress, Gene Expression Omnibus (GEO), and Sequence Read Archive (SRA). The meta information of each sample was manually curated, including virus strain, model, organism, and time point. The expression matrix for each microarray data was downloaded via the GEOquery R package. The matrix was further filtered by removing the probes with expression in only half of the samples. Expression values were normalized using quantile normalization, and log_2_ transformation was applied for each matrix. The probe values were merged based on Entrez Gene ID. The Significance Analysis of Microarrays (SAM) method was used to compute differentially expressed (DE) genes with criteria log_2_ fold change ≥1 or ≤ −1 and false discovery rate (FDR) < 0.05. Gene symbols of other organisms were converted to HUGO gene symbols using the biomaRt package. For RNA-seq datasets, raw sequence data were downloaded from SRA and processed with the TOIL pipeline (Liu et al., 2019; Vivian et al., 2017). EdgeR (Robinson et al., 2010) was used to compute DE genes using the same criteria as used for microarray data, except that log_2_ fold change threshold was set to 0.585 (≈log_2_1.5) for SARS-CoV-2 samples because of fewer DE genes. For the infection group, we enumerated all the comparisons across all time points, and corresponding comparisons were performed in the mock group. The DE genes that were uniquely present in the infection group were selected for further analysis. We also compared DE genes between infection and mock groups at each time point, together with consistently dysregulated genes from the first to last time point.

#### Computation of drug signatures

Drug gene expression profiles have been widely used in our previous studies. Briefly, a full matrix comprising 476,251 signatures and 22,268 genes including 978 landmark genes (as of September 2013) was downloaded from the LINCS website (https://clue.io). The meta-information of the signatures (for example, cell line, treatment duration, treatment concentration) was retrieved via LINCS Application Program Interfaces. The matrix and metadata are now available via GSE92742 in GEO. The signature derived from the comparison of gene expressions between the perturbagen- or vehicle control-treated samples represents gene expression changes upon treatment. We further downloaded the LINCS drug information from the Drug Repurposing Hub. Only small molecules with high-quality gene expression profiles (is_gold = 1, annotated in the meta information) and listed in the Drug Repurposing Hub were further analyzed.

#### Reversal correlation

The computation of Reversal of Gene Expression Score (RGES) and the summarization of RGES (to give the summarized RGES, or sRGES) were detailed elsewhere and recently implemented as a standalone R package (Zeng et al., 2021). In short, we quantified the reversal of disease gene expression as RGES, a measure modified from the connectivity score developed in other studies (Sirota et al., 2011; Subramanian et al., 2017). To compute RGES, we first ranked genes based on their expression values in each drug profile. An enrichment score for each set of up- and down-regulated disease genes was computed separately using a Kolmogorov–Smirnov-like statistic, followed by merging scores from both sets (up/down). The score is based on the extent to which the provided genes (up or down-regulated disease genes) are located at either the top or bottom of the ranked drug expression profile. One compound might have multiple expression profiles because they were tested in various cell lines, drug concentrations, treatment durations, or occasionally different replicates, resulting in multiple RGES for one disease prediction. We set a reference condition (i.e., concentration of 10 μM and treatment duration of 24 h) and used a model to estimate a new RGES if the drug profile under the reference condition was not available. We summarized these scores as sRGES without weighting the cell lines. We considered predictions to be insignificant if the maximum of the absolute sRGES is <0.25.

#### Selection of valid infection signatures

Drugs with known *in vitro* activity against any of the three CoVs (i.e., SARS-CoV, MERS-CoV and SARS-CoV-2) served as positive controls to select valid infection signatures (Data S1). When selecting the first version (V1) of valid signatures, we used all positive drugs reported before February 2020. After updating published anti-SARS-CoV-2 hits, the positive drugs were equally distributed to a calibration set and an external test set. Only the calibration set was used to select valid signatures. Qualified signatures should meet the following criteria: (1) derived from CoV infection experiments; (2) the number of DE genes mapped to LINCS was > 50 (not applied to SARS-CoV-2 signature selection because of generally fewer DE genes); (3) the maximum absolute sRGES prediction was > 0.25; (4) the sRGES of positive drugs was enriched at the top (one side Wilcoxon rank-sum test p < 0.05, FDR < 0.25); (5) the sRGES and the average EC_50_ value of positive drugs were highly correlated (Spearman Rho >= 0.4, p < 0.05; not applied to SARS-CoV-2 signature selection because of the highly varied experimental settings).

#### Quantifying the separation of patient and control samples using a subset of transcriptome

Transcripts per million (TPM) values were used to quantify the transcription of *N* selected genes (e.g., CoV signature genes) in every sample. For a cohort with *M*_1_ patient samples and *M*_2_ control samples (from healthy donors or patients diagnosed with other diseases), an *N* ∗ (*M*_1_ + *M*_2_) matrix was transformed using PCA. Then the first and second PCs were used as input features to fit a linear discriminant analysis (LDA) model. The fitted model assigned a predicted label of “patient” or “control” to each sample. Finally, to quantify the separation, we calculated the balanced accuracy score based on the true label and predicted label of each sample in this cohort.

#### Dose-response curve (DRC) analysis by immunofluorescence

All compounds were purchased from MedChemExpress (Monmouth Junction, NJ) and dissolved in DMSO. Six SARS-CoV-2 variants A, alpha, beta, gamma, delta and kappa (βCoV/KOR/KCDC03/2020, hCoV-19/Korea/KDCA51463/2021, hCoV-19/Korea/KDCA55905/2021, hCoV-19/Korea/KDCA95637/2021, hCoV-19/Korea/KDCA119861/2021, hCoV-19/Korea/KDCA105288/2021, respectively) were provided by Korea Disease Control and Prevention Agency (KDCA) and was propagated in Vero cells. Viral titers were determined by plaque assays in Vero cells. All experiments using SARS-CoV-2 were performed at Institut Pasteur Korea in compliance with the guidelines of the Korea National Institute of Health (KNIH), using enhanced Biosafety Level 3 (BSL-3) containment procedures in laboratories approved for use by the Korea Centers for Disease Control and Prevention (KCDC). Ten-point DRCs were generated for each drug. Vero cells were seeded at 1.2 × 10^4^ cells per well in DMEM, supplemented with 2% FBS and 1X Antibiotic-Antimycotic solution (Gibco/Thermo Fisher Scientific, Waltham, MA, USA) and Calu-3 cells were seeded at 2.0 × 10^4^ cells per well in EMEM, supplemented with 20% FBS, 1X MEM-NEAA (Gibco) and 1X Antibiotic-Antimycotic solution (Gibco) in black, 384-well, μClear plates (Greiner Bio-One, Kremsmünster, Austria), 24 h prior to the experiment. Ten-point DRCs were generated, with compound concentrations ranging from 0.003–50 μM. For viral infection, plates were transferred into the BSL-3 containment facility and SARS-CoV-2 was added at a multiplicity of infection (MOI) of 0.025 or 0.1 for Vero and Calu-3 cells, respectively. The cells were fixed at 24 hpi with 4% PFA and analyzed by immunofluorescence. The acquired images were analyzed using Columbus software (Perkin Elmer, Waltham, MA, USA) to quantify cell numbers and infection ratios, and antiviral activity was normalized to positive (mock) and negative (0.5% DMSO) controls in each assay plate. DRCs were generated in Prism7 (GraphPad, San Diego, CA, USA) software, with Dose-response-inhibition nonlinear regression analysis. IC_50_ and CC_50_ values were measured in duplicates. Mean values of independent duplicate experiments were used for analysis. Each assay was controlled by the Z′-factor and the coefficient of variation in percent (%CV).

#### RNA-seq sample preparing and data processing

Calu-3 cells were seeded onto a 12-well plate 5x10^5^ cells/well in Eagle’s Minimum Essential Medium (EMEM, ATCC), supplemented with 20% heat-inactivated fetal bovine serum (FBS), 1X MEM-NEAA (Gibco) and 1X Antibiotic-Antimycotic solution (Gibco), and maintained at 37°C with 5% CO_2_ for 24 h. The following day Calu-3 cells were treated with either DMSO or IMD-0354 for 30 min prior to SARS-CoV-2 infection. Then the cells were infected with 0.1 MOI SARS-CoV-2 lineage A (βCoV/KOR/KCDC03/2020). Total RNA from the infected cells was isolated 24 h post-infection using MagMAX^TM^ mirVana^TM^ Total RNA Isolation Kit (ThermoFisher, A27828) following the manufacturer’s protocol.

RNA samples from control, infection and treatment samples were prepared using the KAPA RNA HyperPrep Kit and paired-end sequencing was performed in Illumina NextSeq500. The ribosomal reduction RNA-seq approach was used to capture the host and viral RNAs. Sequencing reads were mapped on the human Hg38 transcriptome with the ENSEMBL GRCh38.p3 annotation using STAR aligner (Dobin et al., 2013) to capture the number of reads mapped to individual genes. Mapped reads were used to compute the DE genes using the edgeR package (Robinson et al., 2010) implemented in the OCTAD package (Zeng et al., 2021). The genes with criteria log_2_ fold change ≥1 or ≤ −1 with FDR <0.05 were defined as DE genes. Additionally, to compare the viral genes among each other, the number of reads mapped was normalized with the gene length and total reads. One sample with exceptional high viral gene expression in the treatment group was excluded from the DE analysis.

For viral detection, we started with the unmapped reads left from the human transcriptome mapping to align on the 11406 viral genomes. Since more than 99% of reads were mapped on the SARS-CoV-2 genome, we extracted the reads mapped on the SARS-CoV-2 genome and quantified the reads mapped on each viral gene. Further, raw read counts of each viral gene were normalized with total read counts and gene length. Two sides Wilcox rank sums test was performed to compare viral gene expression in different samples. The p-value cut-off ≤ 0.05 was used to identify the significant difference between the two groups.

#### RNA isolation, cDNA synthesis, and real-time quantitative PCR (RT-qPCR)

Calu-1 cells were treated with IMD-0354 (5 μM) alone or IMD-0354 (5 μM) plus BX795 (5 μM) for 24 h. Then total RNA was isolated from Calu-1 cells using the RNA extraction reagent (Vazyme, Nanjing, China; R401-01). The cDNA was synthesized using the HiScript II Q RT SuperMix (Vazyme, R223-01) according to the manufacturer’s instructions. RT-qPCR was performed using ChamQ SYBR qPCR Master Mix (Vazyme, Q331-02) in CFX96TM Real Time PCR Detection System (Bio-Rad, Shanghai, China). The profile of thermal cycling consisted of initial denaturation at 95°C for 30 s, and 40 cycles at 95°C for 5 s and 60°C for 30 s. All the primer sequences used in this study are as follows: human ACTB forward: catgtacgttgctatccaggc, human ACTB reverse: ctccttaatgtcacgcacgat; human IFNB forward: cagcatctgctggttgaaga, human IFNB reverse: cattacctgaaggccaagga; human CXCL10 forward: ccacgtgttgagatcattgct, human CXCL10 reverse: tgcatcgattttgctcccct; human IL6 forward: ttcggtccagttgccttctc, human IL6 reverse: tacatgtctcctttctcagggc. Following this protocol, IFNB, CXCL10 and IL6 expression could be induced by 5 μM diABZI (the positive control, a Sting agonist; Table S4). In this section, the Calu-1 cells were used instead of Calu-3, only because the latter were out of our stock. Given the urgent situation, we proceeded with Calu-1, a male non-small cell lung cancer cell line similar with Calu-3.

#### Calculation of CoV meta-signatures

The valid signature datasets were used in the MetaIntegrator package (Haynes et al., 2016), and their effect size was calculated for the disease. The effect size cutoff of ≤ −1 or ≥1 with adjusted p-value ≤ 0.01 was used to filter the genes with significant effect size.

#### Pilot drug screening by cell-based assay

Vero E6 cells [CRL:1586, ATCC] were grown in Eagle’s minimal essential medium (EMEM) supplemented with penicillin (100 units/mL), streptomycin (100 μg/mL), and 10% fetal bovine serum (FBS). SARS-CoV-2 (US_WA-1 isolate), the 3^rd^ passage in Vero E6 cells from the original CDC (Atlanta) material and sequence confirmed, was used throughout the pilot screening. The titer of the viral stock was 7.5 × 10^7^ 50% tissue culture infectious doses (TCID_50_)/mL. All experiments involving infectious virus were conducted at the University of Texas Medical Branch in an approved biosafety level 3 laboratory. A slightly modified Vero E6-based standard micro-neutralization assay was used to rapidly evaluate the drug efficacy against SARS-CoV-2 infection. Briefly, confluent Vero E6 cells grown in 96-wells microtiter plates were pre-treated with serially 2-folds diluted individual drugs for 2 h before infection with 100 infectious SARS-CoV-2 particles in 100 μL EMEM supplemented with 2% FBS. Vero E6 cells treated with parallelly diluted dimethyl sulfoxide (DMSO) with or without virus were included as positive and negative controls, respectively. After cultivation at 37°C for 4 days, individual wells were observed under the microcopy for the status of virus-induced formation of cytopathic effect. The efficacy of individual drugs was calculated and expressed as the lowest concentration capable of completely preventing virus-induced CPE in 100% of the wells. The toxicity to the treated cells was assessed by observing floating cells and altered morphology of adhered Vero E6 cells in wells under the microcopy. All compounds were ordered from Selleckchem (Houston, TX) or Cayman Chemical (Ann Arbor, MI). All compounds were dissolved in 100% DMSO as 10 mM stock solutions and diluted in culture media.

#### Drug profile heatmap visualization

We used the summarized profiles across different cellular contexts and treatment durations, as previously published (Xing et al., 2021). Briefly, the level-5 profiles derived from the comparison of gene expressions between the drug- or vehicle control-treated samples represent gene expression changes upon treatment. Only 978 landmark genes were included. As one drug could be profiled under different concentrations, treatment durations, and cellular contexts, for a specific drug, we took the median LINCS z-scores of its profiles measured at 10 μM of treatment, regardless of the time and cellular context.

#### Gene Ontology enrichment analysis

We evaluated gene sets in the three categories of Gene Ontology, i.e., Biological Process, Molecular Function and Cellular Component. Each category was calculated separately. The gene universe (background) was defined as a union of DE genes in all valid CoV signatures and all genes annotated in a specific category. Then we used Fisher exact test to calculate the p value of a gene set enrichment in the up- or down-regulated genes from a valid CoV signature. Benjamini-Hochberg procedure was performed to calculate the FDR of each gene set within a category. For improved visualization, the FDR values were transformed by -log10 for up-regulation enrichment and by +log10 for down-regulation enrichment, and we took the one with a larger absolute value as the final direction. Finally, gene sets enriched in more than half of the valid signatures (v1) or no less than 10 of the valid signatures (v2) were inspected and removed redundant gene sets for biological interpretation.

#### MoA query among perturbagen transcriptomic profiles

Similar to the idea of “Connectivity Map” (Subramanian et al., 2017), the query input was the names and directions of the DE genes induced by a treatment, e.g., IMD-0354 induced up- and down-regulated genes compared with the control group. The searched perturbagen (a gene knock-down or over-expression) database was the LINCS L1000 high-quality dataset (is_gold = 1, annotated in the meta information, GSE92742 and GSE70138). Before searching, we summarized a robust gene expression change profile for each perturbagen by taking the median *Z* score of each landmark gene across different cell types and treatment times. Of note, only consensus gene knockdown signatures (CGS) were used unless no “CGS” profiles were measured for a given shRNA perturbagen. This resulted in 4,370 different shRNA and 2,878 different over-expression profiles after merging, with each profile containing expression z-scores of 978 landmark genes. Then the correlation between the query gene sets (overlapped with the LINCS landmark gene set) and each perturbagen was evaluated by RGES and Wilcox rank sums test. For the Wilcox rank sums test, one-sided p values of up- and down-regulated genes were first calculated separately then merged as 1 – (1 – *P*_up_) ∗ (1 – *P*_down_) followed by Benjamini–Hochberg FDR correction. A positive RGES and FDR_mimic_< 0.05 indicate that the perturbagen and the query treatment share similar MoA, whereas a negative RGES and FDR_reversal_< 0.05 indicate the opposite MoA.

We further validated a few hits using another dataset GSE161664 with the whole transcriptome profiled. Raw FASTQ files for SRP292952 (https://trace.ncbi.nlm.nih.gov/Traces/sra/?study=SRP292592) were downloaded from public database NCBI SRA (https://www.ncbi.nlm.nih.gov/sra). Data were processed using RSEM (Li and Dewey, 2011) 1.3.1 + STAR (Dobin et al., 2013) 2.6.1 pipeline. The RNA-seq processing code is available at GitHub (https://github.com/Bin-Chen-Lab/chenlab_toil). Sample metadata were obtained from GEO (GSE161664). Log_2_ transformed (addition of pseudocount 1) read count values as gene expression measures were employed to calculate log_2_ fold changes between treated (e.g. IFN-β) and control groups using the diffExp function in the OCTAD R package (Zeng et al., 2021). The correlation between the query gene sets and each perturbagen was calculated in the same way as mentioned above, except that the query input was not reduced.

#### RNA isolation, cDNA synthesis, and real-time quantitative PCR (RT-qPCR)

Human myeloid leukemia mononuclear cells (THP-1) (ATCC, TIB-202) were cultured in RPMl Medium 1640 (Gibco, 61870–036) supplemented with 10% FBS (Gibco, 10099-141) and 0.05 mM β-mercaptoethanol (Gibco, 21985). THP-1 cells were differentiated into macrophage-like cells (THP-1-derived macrophages) by incubation in the presence of PMA (100 nM) for 48 h. THP-1-derived macrophages were treated with IMD-0354 for 8 h, then total RNA was isolated using RNA extraction reagent (Vazyme, R401-01). cDNA was synthesized using the HiScript III RT SuperMix for qPCR (+gDNA wiper) (Vazyme, R323-01) according to the manufacturer’s instructions. RT-qPCR was performed using ChamQ SYBR qPCR Master Mix (Vazyme, Q331-02) in CFX96TM RealTime PCR Detection System (BioRad, Shanghai, China). The profile of thermal cycling consisted of initial denaturation at 95°C for 30 s, and 40 cycles at 95°C for 5 s and 60°C for 30 s. The specificity of primers was examined by melting curve analysis and agarose gel electrophoresis of PCR products. All the primer sequences used in this study are as follows: human ACTB forward: catgtacgttgctatccaggc, human ACTB reverse: ctccttaatgtcacgcacgat; human IFNB1 forward: cagcatctgctggttgaaga, human IFNB1 reverse: cattacctgaaggccaagga; human CXCL10 forward: ccacgtgttgagatcattgct, human CXCL10 reverse: tgcatcgattttgctcccct human IL6 forward: ttcggtccagttgccttctc, human IL6 reverse: tacatgtctcctttctcagggc.

#### Drug co-treatment and dose-response curve (DRC) analysis by immunofluorescence

Ten-point DRCs were generated for each drug with co-treatment of BX-795 or ruxolitinib. Calu-3 cells were seeded at 2.0 × 10^4^ cells per well in EMEM, supplemented with 20% FBS, 1X MEM-NEAA (Gibco) and 1X Antibiotic-Antimycotic solution (Gibco) in black, 384-well, μClear plates (Greiner Bio-One, Kremsmünster, Austria), 24 h prior to the experiment. Ten-point DRCs were generated for IMD-0354 and remdesivir, with compound concentrations ranging from 0.003–50 μM. Then 0.5 or 5μM of BX-795 or ruxolitinib was added to the DRCs. For viral infection, plates were transferred into the BSL-3 containment facility and SARS-CoV-2 was added at a multiplicity of infection (MOI) of 0.1. The cells were fixed at 24 hpi with 4% PFA and analyzed by immunofluorescence. The acquired images were analyzed using Columbus software (Perkin Elmer, Waltham, MA, USA) to quantify cell numbers and infection ratios, and antiviral activity was normalized to positive (mock) and negative (0.5% DMSO) controls in each assay plate. DRCs were generated in Prism7 (GraphPad, San Diego, CA, USA) software, with Dose-response-inhibition nonlinear regression analysis. IC_50_ and CC_50_ values were measured in duplicates. Mean values of independent duplicate experiments were used for analysis. Each assay was controlled by the Z′-factor and the coefficient of variation in percent (%CV).

### Quantification and statistical analysis

All analyses were conducted in R (v3.5.1) or Python (v3.7) programming language. The ggplot2, pheatmap, matplotlib, and seaborn packages were used for data visualization. The Student’s *t* test was performed for normally distributed data, and Wilcoxon rank-sum test was used for other types of data to compute the p value. PCA, LDA, and accuracy calculation were calculated with the Sci-kit Learn package in Python.

## Data Availability

The authors declare that all data used in this study are available within the article and its supplemental information files. Additional Supplemental Items are available from Mendeley Data: https://doi.org/10.17632/wg8mwn4c9j.1 Other specific files can be provided by the corresponding author upon reasonable request. The code is available at GitHub (https://github.com/Bin-Chen-Lab/wars). RNA-seq data for control, SARS-CoV-2 infected, and IMD-0354 treated samples are available at GEO: GSE187420.

## Supplemental information

Document S1. Figures S1–S8 and Tables S1–S5

Data S1. Published anti-coronavirus compounds, their efficacy, cytotoxicity, references, etc, related to Figure 1

Data S2. Meta-information of RNA-seq samples used to create CoV signatures, related to Figure 2

Data S3. Evaluation of raw CoV signatures V1, related to Figure 3

Data S4. Selected (valid) CoV signatures V1 and the merged meta-signature, related to Figure 3

Data S5. Ranks of all repurposed drug candidates based on their ability of reversing CoV valid signatures V1, related to Figure 3

Data S6. GO enrichment analysis of the valid CoV signatures, related to Figure 3

Data S7. Selected (valid) CoV signatures V2 and the merged meta-signature, related to Figure 3

Data S8. Patient or pre-clinical sample groups for CoV meta-signature validation, related to Figure 4

Data S9. Ranks of all repurposed drug candidates based on their ability of reversing CoV valid signatures V2, related to Figure 5

Data S10. Whole-transcriptomic gene log2 fold changes of our RNA-seq data, interferons treated airway cells, and ruxolitinib treated airway cells, related to Figure 6 and Figure 7

Data S11. IMD-0354 treatment vs control log2 fold changes and averaged log2 fold changes of CoV valid signatures, related to Figure 7

Data S12. GO enrichment analysis of DE genes induced by IMD-0354 treatment, related to Figure 7

Data S13. RGES and Wilcoxon rank-sum test results of LINCS perturbagens aligned to IMD-0354 RNA-seq DE genes, together with significant virus-hijacked proteins, related to Figure 7

Data S14. Landmark gene expression change induced by selected perturbagens aligned to IMD-0354 RNA-seq DE genes, related to Figure 7
