# Supplementary material for: Deciphering COVID-19 host transcriptomic complexity and variations for therapeutic discovery against new variants
Source: iScience. 2022 Sep 3;25(10):105068. doi: 10.1016/j.isci.2022.105068 (PMC9439871; doi:10.1016/j.isci.2022.105068)
Supplement: Document S1. Figures S1–S8 and Tables S1–S5 [file mmc1.pdf]

iScience, Volume *25*

Supplemental information

Deciphering COVID-19 host transcriptomic complexity and variations for therapeutic discovery against new variants

Jing Xing, Rama Shankar, Meehyun Ko, Keke Zhang, Sulin Zhang, Aleksandra Drelich, Shreya Paithankar, Eugene Chekalin, Mei-Sze Chua, Surender Rajasekaran, Chien-Te Kent Tseng, Mingyue Zheng, Seungtaek Kim, and Bin Chen

Table S1. Published COVID-19 transcriptomic signatures. Related to Figure 1.

COVID-19 Signatures	PMID	Journal	Note
Le_gut_organoids	34112877	Sci Rep	
Le_patients	34112877	Sci Rep	
Blanco-Melo_Calu3	32416070	Cell	
Blanco-Melo_A549_1	32416070	Cell	A549
Blanco-Melo_A549_2	32416070	Cell	A549-ACE2_lowMOI
Blanco-Melo_A549_3	32416070	Cell	A549-ACE2_highMOI
Blanco-Melo_NHBE	32416070	Cell	
Blanco-Melo_patients	32416070	Cell	
Winkler_mice_D2	32839612	Nat Immunol	
Winkler_mice_D4	32839612	Nat Immunol	
Winkler_mice_D7	32839612	Nat Immunol	
Lieberman_patients_1	32898168	PLoS Biol	Covid_vs_ctrl
Lieberman_patients_2	32898168	PLoS Biol	High_viral_load_vs_low
Mick_patients	33203890	Nat Commun	
Overmyer_patients	33096026	Cell Syst	
Gill_patients	33306162	Intensive Care Med Exp	
Yuan_Caco2	33727703	Nature	
Singh_patients	34586723	Clin Transl Med	

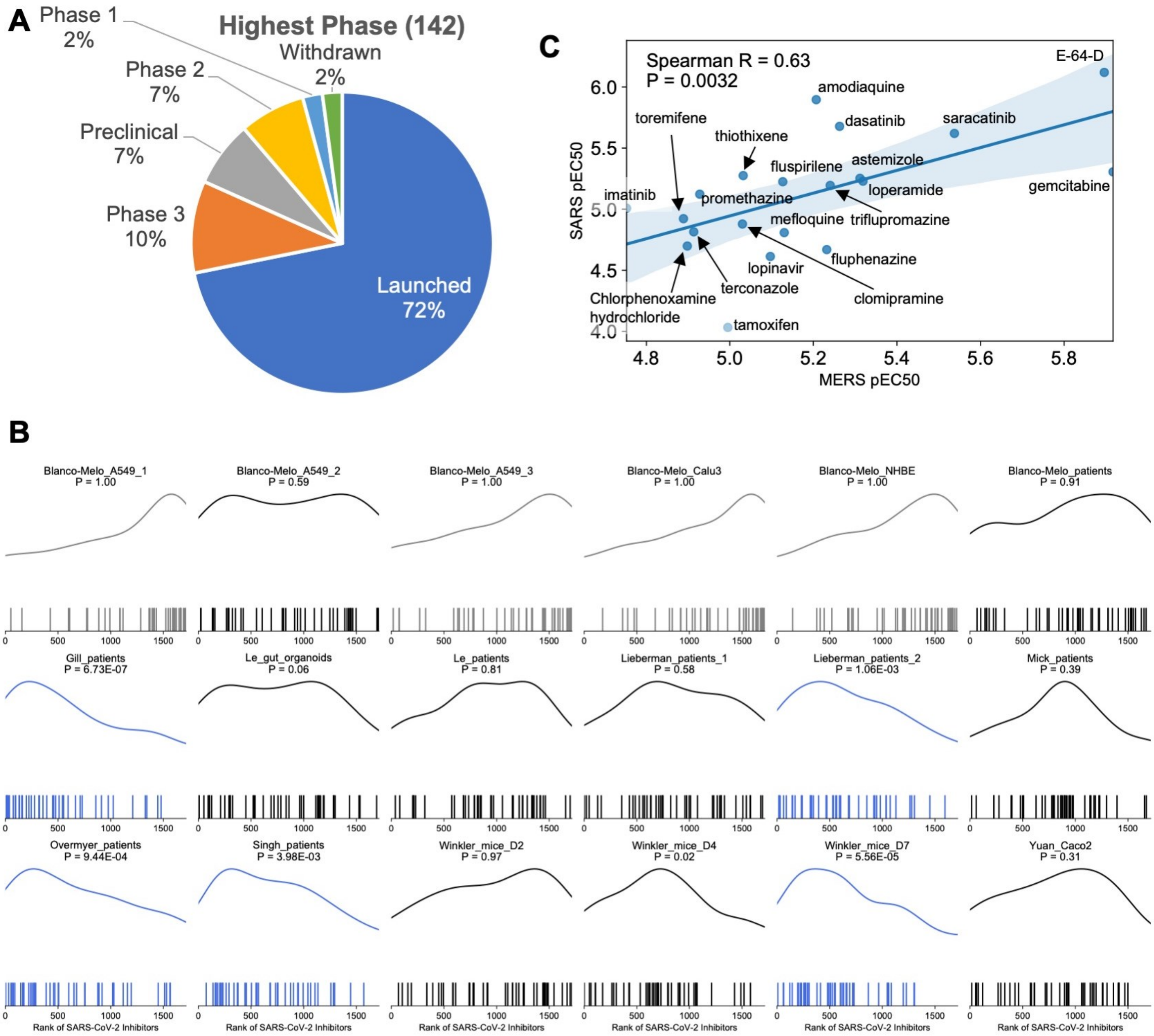

Figure S1. Summary of published CoV inhibitors and drug prediction performance of COVID-19 signatures. A, Pie chart of the highest phases of published CoV inhibitors. **B**, Enrichment of positive hits for each of the COVID-19 signatures. Grey: negative correlation; black: not significant; blue: positive correlation. **C**, Correlation between compounds' anti-SARS activity and anti-MERS activity. Related to Figure 1.

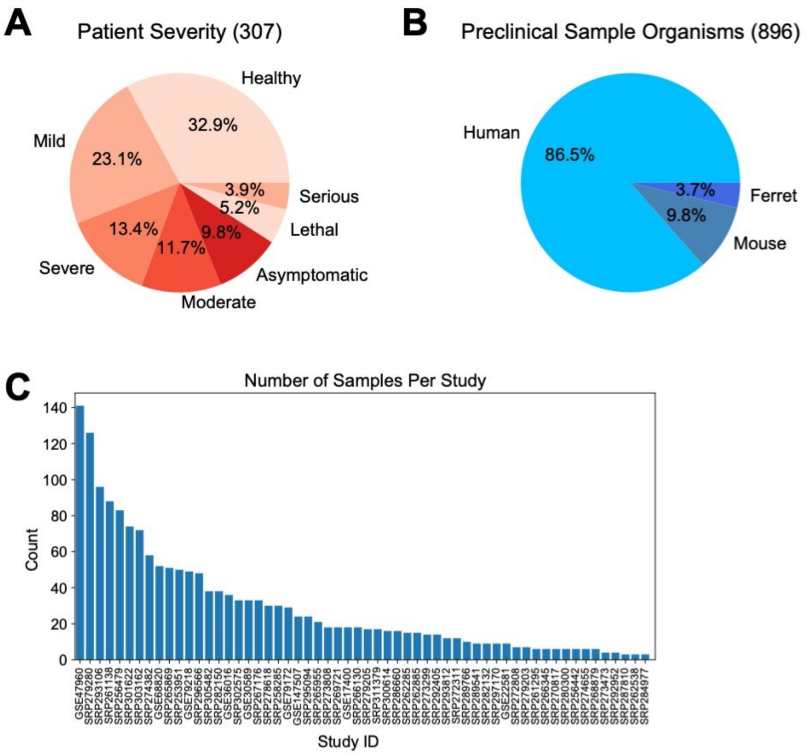

Figure S2. Summary of CoV-induced host transcriptomic change datasets, including patient severities (A), different species (B), and sample size per dataset (C). Related to Figure 2.

Table S2. Data sets used for disease signature creation (Version 1). Related to Figure 3.

S. No.	Accession	Platform	Disease/ Infection	Organism	Model	Time points	Number of samples	PMID
1	GSE17400	GPL570	MOCK, DOHV and SARS	Homo sapiens	Calu-3	3	27	20090954
2	GSE30589	GPL570	SARS and MOCK	Homo sapiens	Vero E6, Vero E6 DeltaE, MA-104 and MA-104 DeltaE	4	33	22028656
3	GSE45042	GPL6480	MOCK and EMC	Homo sapiens	Calu-3	6	33	23631916<br>24846384
4	GSE47960	GPL6480	MOCK, SARS, H1N1, SARS-BatSRBD and SARS-dORF6	Homo sapiens	HAE	11	163	23935999
5	GSE79218	GPL13497	MOCK and MERS	Homo sapiens	MMVE001	5	49	
6	GSE79172	GPL13497	MOCK and MERS	Homo sapiens	MDC001	5	29	28830941
7	GSE22581	GPL3738	MOCK and SARS	Canis lupus	Ferret Lung	3	9	21035159

8	GSE36016	GPL7202	MOCK and SARS	Mus musculus	lung WT, lung IFNAR1 and lung STAT	3	36	
9	GSE68820	GPL7202	MOCK and SARS	Mus musculus	lung wt and lung TLR3	3	52	26015500
10	GSE30351	GPL6480	JFH1	Homo sapiens	Huh7	2	7	
11	GSE71063	GPL570	HIV	Homo sapiens	patients	2	40	26935044

Table S3. Positive control drugs with known activity against MERS-CoV/SARS-CoV/SARS-CoV-2 (Version 1). Related to Figure 3.

Name	MERS EC50 μM	SARS EC50 μM	COVID-19 EC50 μM	MoA	PMID
Amodiaquine	6.21	1.27	NA*	Antiparasitic agent	32006468
Astemizole	4.88	5.59	NA	Neurotransmitter inhibitor	32006468
Bisindolylmaleimide-ix	Inhibition 74% @ 10uM	NA	NA	PKC inhibitor	25653449
Bufalin	Inhibition > 90% @ 10 nM	NA	NA	Inhibit MERS-CoV entry by blocking clathrin-mediated endocytosis	25653449
Chloroquine	3	NA	1.13	Endosomal acidification inhibitor	32006468
Chlorpromazine	4.9	NA	NA	Neurotransmitter inhibitor	32006468
Clomipramine	9.33	13.23	NA	Neurotransmitter inhibitor	32006468
Dasatinib	5.46	2.1	NA	ABL1 inhibitor	32006468
Disulfiram	NA	NA	NA	MERS-CoV PL-pro inhibitor	32006468
Emetine	0.34	NA	NA	Inhibits RNA, DNA and protein synthesis	32006468
Fluphenazine	5.86	21.43	NA	Neurotransmitter inhibitor	32006468
Fluspirilene	7.47	5.96	NA	Neurotransmitter inhibitor	32006468
Gemcitabine	1.21	4.95	NA	DNA metabolism inhibitor	32006468
GW-5074	Inhibition 52% @ 10uM	NA	NA	Raf inhibitor	25653449
Imatinib	17.68	9.82	NA	ABL1 inhibitor	32006468
Loperamide	4.8	5.9	NA	Antidiarrheal opioid receptor agonist	32006468
Lopinavir	8	24.4	NA	HIV-1 inhibitor	32006468
Mefloquine	7.41	15.55	NA	Antiparasitic agent	32006468
Mg-132	NA	NA	NA	Cys-protease m-calpain inhibition	22787216
Monensin	3.27	NA	NA	Antibacterial	32006468
Mycophenolate-mofetil	1.54	NA	NA	Immune suppressant, antineoplastic, antiviral	32006468
Niclosamide	NA	2	NA	Antiparasitic agent	15215127
Nitazoxanide	NA	NA	2.12	Antiprotozoal, type I IFN inducer	32020029
Ouabain	Inhibition 70% @ 50 nM	NA	NA	Inhibit MERS-CoV entry by blocking clathrin-mediated endocytosis	25653449
Penciclovir	NA	NA	95.96	Inhibition of virus DNA synthesis	32020029
Phenazopyridine	1.93	NA	NA	Analgesic	32006468
Promethazine	11.8	7.54	NA	Neurotransmitter inhibitor	32006468
Pyrvinium-pamoate	1.84	NA	NA	Anthelmintic	32006468
Ribavirin	NA	NA	109.5	Ribonucleic analog	32020029
Ritonavir	24.9	NA	NA	HIV protease inhibitor	31924756

Name	MERS EC50 μM	SARS EC50 μM	COVID-19 EC50 μM	MoA	PMID
Saracatinib	2.9	2.4	NA	Src family of tyrosine kinases inhibitor	32006468
SB-203580	Inhibition 45% @ 10uM	NA	NA	p38 MAPK inhibitor	25653449
Selumetinib	Inhibition >= 95% @ 10uM	NA	NA	MEK1, ERK1/2 inhibitor	25653449
Sirolimus	Inhibition 61% @ 10uM	NA	NA	MTOR inhibitor	25653449
Tamoxifen	10.11	92.88	NA	Estrogen receptor inhibitor	32006468
Terconazole	12.2	15.32	NA	Sterol metabolism inhibitor	32006468
Thiothixene	9.29	5.31	NA	Neurotransmitter inhibitor	32006468
Toremifene	12.91	11.96	NA	Estrogen receptor inhibitor	32006468
Trametinib	Inhibition >= 95% @ 10uM	NA	NA	MEK1/2 inhibitor	25653449
Triflupromazine	5.75	6.39	NA	Neurotransmitter inhibitor	32006468
U-0126	Inhibition 51% @ 10uM	NA	NA	MEK1/2 inhibitor	25653449

* “NA” indicates not available.

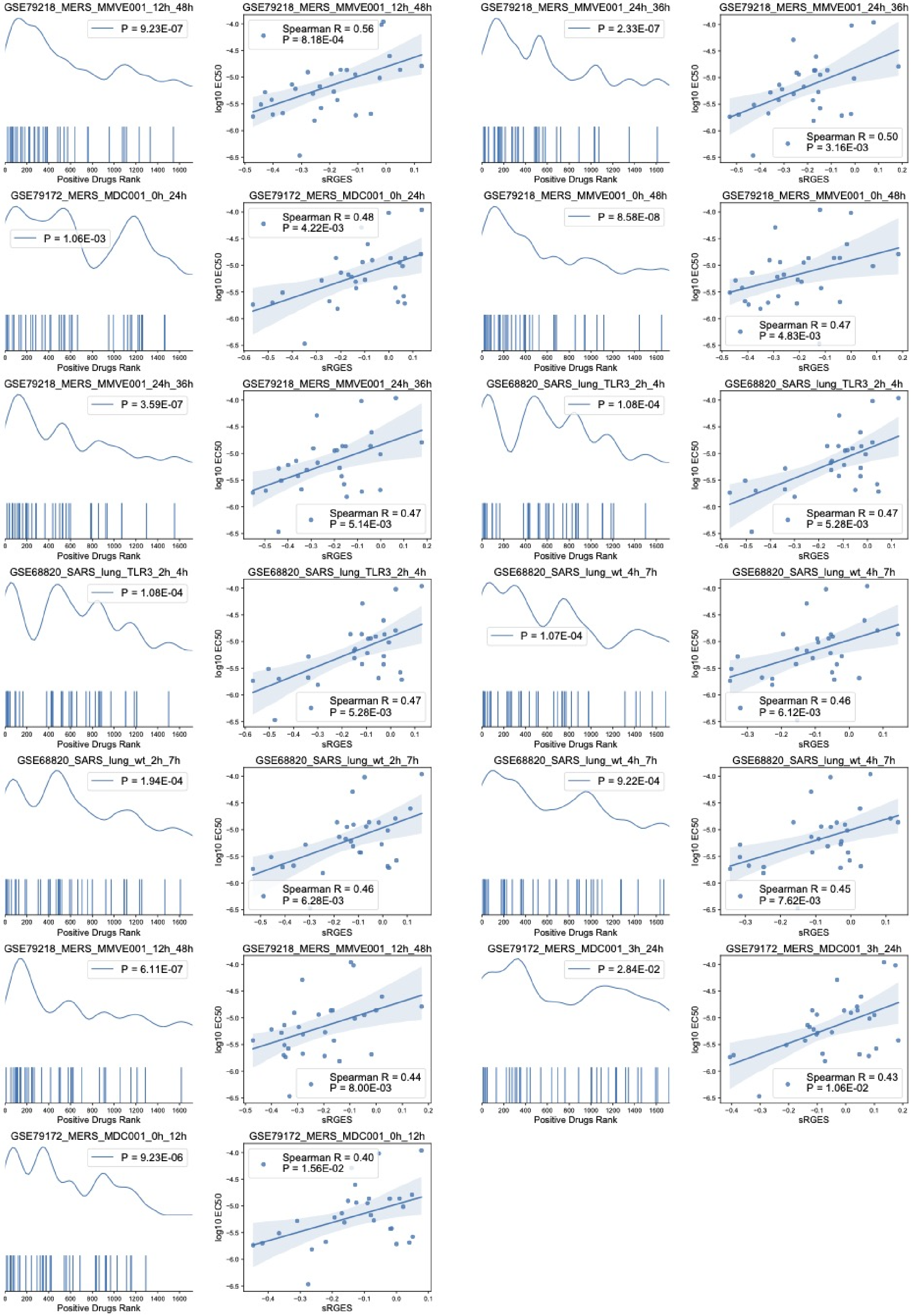
GSE79218_MERS_MMVE001_12h_48h
P = 9.23E-07
Positive Drugs Rank
GSE79218_MERS_MMVE001_12h_48h
Spearman R = 0.56
P = 8.18E-04
log10 EC50
sRGES
GSE79218_MERS_MMVE001_24h_36h
P = 2.33E-07
Positive Drugs Rank
GSE79218_MERS_MMVE001_24h_36h
Spearman R = 0.50
P = 3.16E-03
log10 EC50
sRGES
GSE79172_MERS_MDC001_0h_24h
P = 1.06E-03
Positive Drugs Rank
GSE79172_MERS_MDC001_0h_24h
Spearman R = 0.48
P = 4.22E-03
log10 EC50
sRGES
GSE79218_MERS_MMVE001_0h_48h
P = 8.58E-08
Positive Drugs Rank
GSE79218_MERS_MMVE001_0h_48h
Spearman R = 0.47
P = 4.83E-03
log10 EC50
sRGES
GSE79218_MERS_MMVE001_24h_36h
P = 3.59E-07
Positive Drugs Rank
GSE79218_MERS_MMVE001_24h_36h
Spearman R = 0.47
P = 5.14E-03
log10 EC50
sRGES
GSE68820_SARS_lung_TLR3_2h_4h
P = 1.08E-04
Positive Drugs Rank
GSE68820_SARS_lung_TLR3_2h_4h
Spearman R = 0.47
P = 5.28E-03
log10 EC50
sRGES
GSE68820_SARS_lung_TLR3_2h_4h
P = 1.08E-04
Positive Drugs Rank
GSE68820_SARS_lung_TLR3_2h_4h
Spearman R = 0.47
P = 5.28E-03
log10 EC50
sRGES
GSE68820_SARS_lung_wt_4h_7h
P = 1.07E-04
Positive Drugs Rank
GSE68820_SARS_lung_wt_4h_7h
Spearman R = 0.46
P = 6.12E-03
log10 EC50
sRGES
GSE68820_SARS_lung_wt_2h_7h
P = 1.94E-04
Positive Drugs Rank
GSE68820_SARS_lung_wt_2h_7h
Spearman R = 0.46
P = 6.28E-03
log10 EC50
sRGES
GSE68820_SARS_lung_wt_4h_7h
P = 9.22E-04
Positive Drugs Rank
GSE68820_SARS_lung_wt_4h_7h
Spearman R = 0.45
P = 7.62E-03
log10 EC50
sRGES
GSE79218_MERS_MMVE001_12h_48h
P = 6.11E-07
Positive Drugs Rank
GSE79218_MERS_MMVE001_12h_48h
Spearman R = 0.44
P = 8.00E-03
log10 EC50
sRGES
GSE79172_MERS_MDC001_3h_24h
P = 2.84E-02
Positive Drugs Rank
GSE79172_MERS_MDC001_3h_24h
Spearman R = 0.43
P = 1.06E-02
log10 EC50
sRGES
GSE79172_MERS_MDC001_0h_12h
P = 9.23E-06
Positive Drugs Rank
GSE79172_MERS_MDC001_0h_12h
Spearman R = 0.40
P = 1.56E-02
log10 EC50
sRGES

Figure S3. SARS/MERS signatures (v1) validation using known active drugs (positive controls). The first and third columns show enrichment density and barcode, with p values shown at upper right. The curve shows enrichment density, and each bar under the curve represents the rank of a positive drug among all the drugs in one prediction. The second and last columns list the correlation between sRGES and EC_{50} (in M, log10 transformed) of the positive controls, with Spearman R and p-value shown. Each point indicates a positive control drug. Related to Figure 3.

Table S4. Efficacy of selected drugs against SARS-CoV-2 and their cytotoxicity (pilot screening). Related to Figure 3.

	MoA	Primary Indication	CPE Preventing[*1] (μM)	Toxic[*2] (μM)	Repeat[*3] CPE Preventing (μM)	Repeat Toxic (μM)
Bortezomib	Proteasome inhibitor	Multiple myeloma	0.05	0.002	> 30	> 30
Puromycin	Protein synthesis inhibitor	Antibiotic	20	0.002	-[*4]	-
Methotrexate	Dihydrofolate reductase inhibitor	Rheumatoid arthritis	0.78	> 25	> 30	30
Methylene-blue	Guanylyl cyclase inhibitor	Methemoglobinemia	20	5	-	-
Tyloxapol	NF-kB pathway inhibitor	Bronchopulmonary secretions with mucus and pus	> 100	20	-	-
Nisoldipine	Calcium channel blocker	Hypertension	6.25	0.2	-	-
Nvp-bez235	PI3K/MTOR inhibitor	Advanced Solid Malignancies	1	1	> 30	7.5
Fluvastatin	HMGCR inhibitor	Hypercholesterolemia	3.125	0.2	-	-
Alvocidib	CDK inhibitor	Acute myeloid leukemia	0.05	0.002	> 30	3.75
Dasatinib	SRC/ABL kinase inhibitor	Leukemia	3.125	1.6	-	-
Chloroquine[*5]	Endosomal acidification inhibitor	Malaria	-	-	15	> 30

[*1], the lowest concentration of a drug to prevent CPE.

[*2], the lowest concentration of a drug showing cytotoxicity.

[*3], when repeated, drug treatment time was shorter (2 hours) than the previous test (3 hours).

[*4], not tested.

[*5], the positive control of this assay.

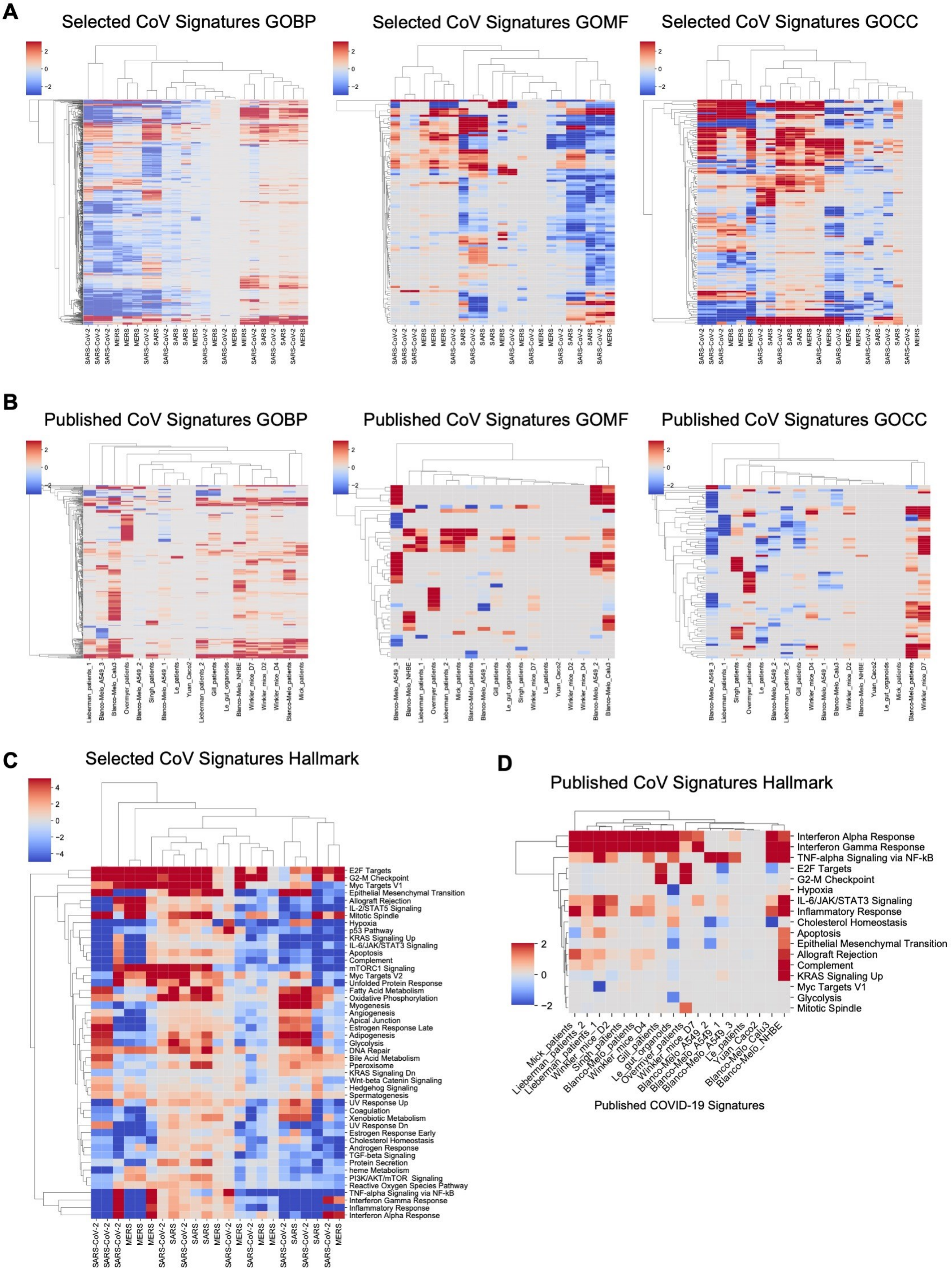

A
Selected CoV Signatures GOBP
Selected CoV Signatures GOMF
Selected CoV Signatures GOCC
B
Published CoV Signatures GOBP
Published CoV Signatures GOMF
Published CoV Signatures GOCC
Lieberman_patients_1
Blanco-Melo_A549_3
Blanco-Melo_Calu3
Overmyer_patients
Blanco-Melo_A549_2
Singh_patients
Blanco-Melo_A549_1
Le_patients
Yuan_Caco2
Lieberman_patients_2
Gill_patients
Le_gut_organoids
Blanco-Melo_NHBE
Winkler_mice_D7
Winkler_mice_D2
Winkler_mice_D4
Mick_patients
C
Selected CoV Signatures Hallmark
E2F Targets
G2-M Checkpoint
Myc Targets V1
Epithelial Mesenchymal Transition
Allograft Rejection
IL-2/STAT5 Signaling
Mitotic Spindle
Hypoxia
p53 Pathway
KRAS Signaling Up
IL-6/JAK/STAT3 Signaling
Apoptosis
Complement
mTORC1 Signaling
Myc Targets V2
Unfolded Protein Response
Fatty Acid Metabolism
Oxidative Phosphorylation
Myogenesis
Angiogenesis
Apical Junction
Estrogen Response Late
Adipogenesis
Glycolysis
DNA Repair
Bile Acid Metabolism
Peroxisome
KRAS Signaling Dn
Wnt-beta Catenin Signaling
Hedgehog Signaling
Spermatogenesis
UV Response Up
Coagulation
Xenobiotic Metabolism
UV Response Dn
Estrogen Response Early
Cholesterol Homeostasis
Androgen Response
TGF-beta Signaling
Protein Secretion
heme Metabolism
PI3K/AKT/mTOR Signaling
Reactive Oxygen Species Pathway
TNF-alpha Signaling via NF-kB
Interferon Gamma Response
Inflammatory Response
Interferon Alpha Response
SARS-CoV-2
SARS
MERS
D
Published CoV Signatures Hallmark
Interferon Alpha Response
Interferon Gamma Response
TNF-alpha Signaling via NF-kB
E2F Targets
G2-M Checkpoint
Hypoxia
IL-6/JAK/STAT3 Signaling
Inflammatory Response
Cholesterol Homeostasis
Apoptosis
Epithelial Mesenchymal Transition
Allograft Rejection
Complement
KRAS Signaling Up
Myc Targets V1
Glycolysis
Mitotic Spindle
Mick_patients
Lieberman_patients_2
Lieberman_patients_1
Winkler_mice_D2
Singh_patients
Blanco-Melo_A549_2
Winkler_mice_D4
Gill_patients
Le_gut_organoids
Overmyer_patients
Winkler_mice_D7
Blanco-Melo_A549_1
Blanco-Melo_A549_3
Le_patients
Yuan_Caco2
Blanco-Melo_Calu3
Blanco-Melo_NHBE
Published COVID-19 Signatures

Figure S4. Comparison of enriched pathways in selected CoV signatures (v2) and published COVID-19 signatures. A, GOBP, GOMF, GOCC terms enriched in selected CoV signatures. B, GOBP, GOMF, GOCC terms enriched in published CoV signatures. C, Hallmark pathways enriched in selected CoV signatures. D, Hallmark pathways enriched in published CoV signatures (same as Figure 1B). Color code depicts FDR (A-C) or p-values (D) (Fisher exact test, log10 transformed, inversed for up-regulation), with red representing up-regulation and blue representing down-regulation. Each column indicates a CoV signature, and each row indicates a GO term or pathway. Related to Figure 3.

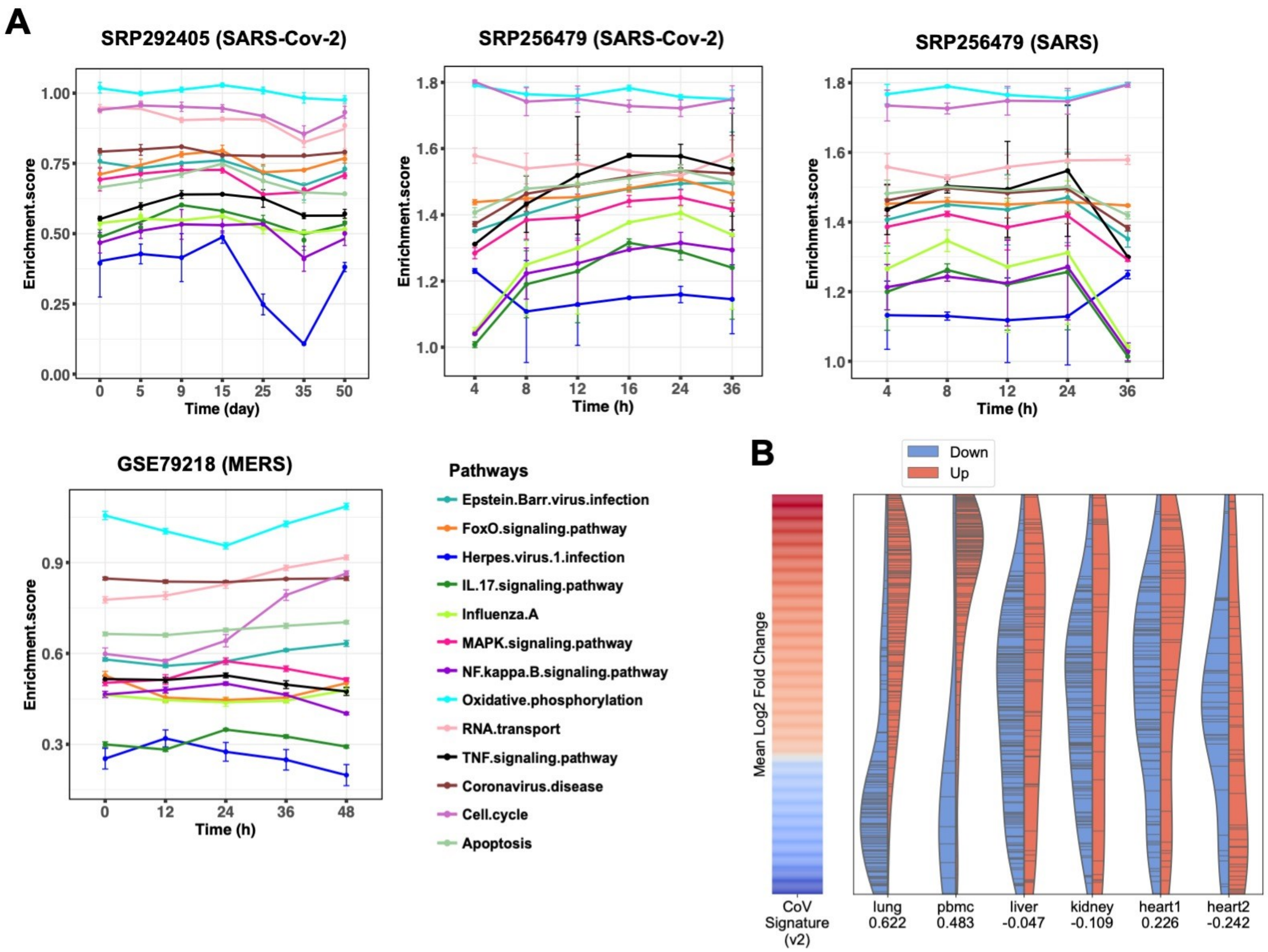

Figure S5. The dynamics and diversity of raw CoV signatures. A, Examples of viral infection related pathway enrichment (ssGSEA) changes over infection time. Dataset IDs and virus types are shown in the panel titles. The enrichment scores were calculated with the GSVA R package. B, Differentially expressed (DE) genes in infected organs aligned to the CoV meta-signature. Red and blue indicate up-regulation and down-regulation, respectively. Horizontal lines inside the density shades mark the positions of organ infection DE genes in the CoV meta-signature. The Spearman correlation between the CoV meta-signature and each organ infection log2 fold changes was annotated under the organ name. Related to Figure 3.

A

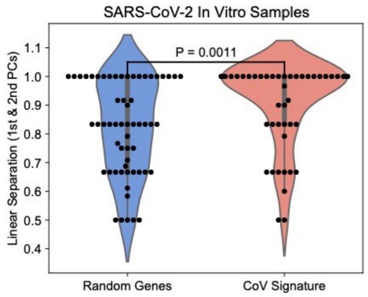
SARS-CoV-2 In Vitro Samples
P = 0.0011
Linear Separation (1st & 2nd PCs)
Random Genes
CoV Signature

B

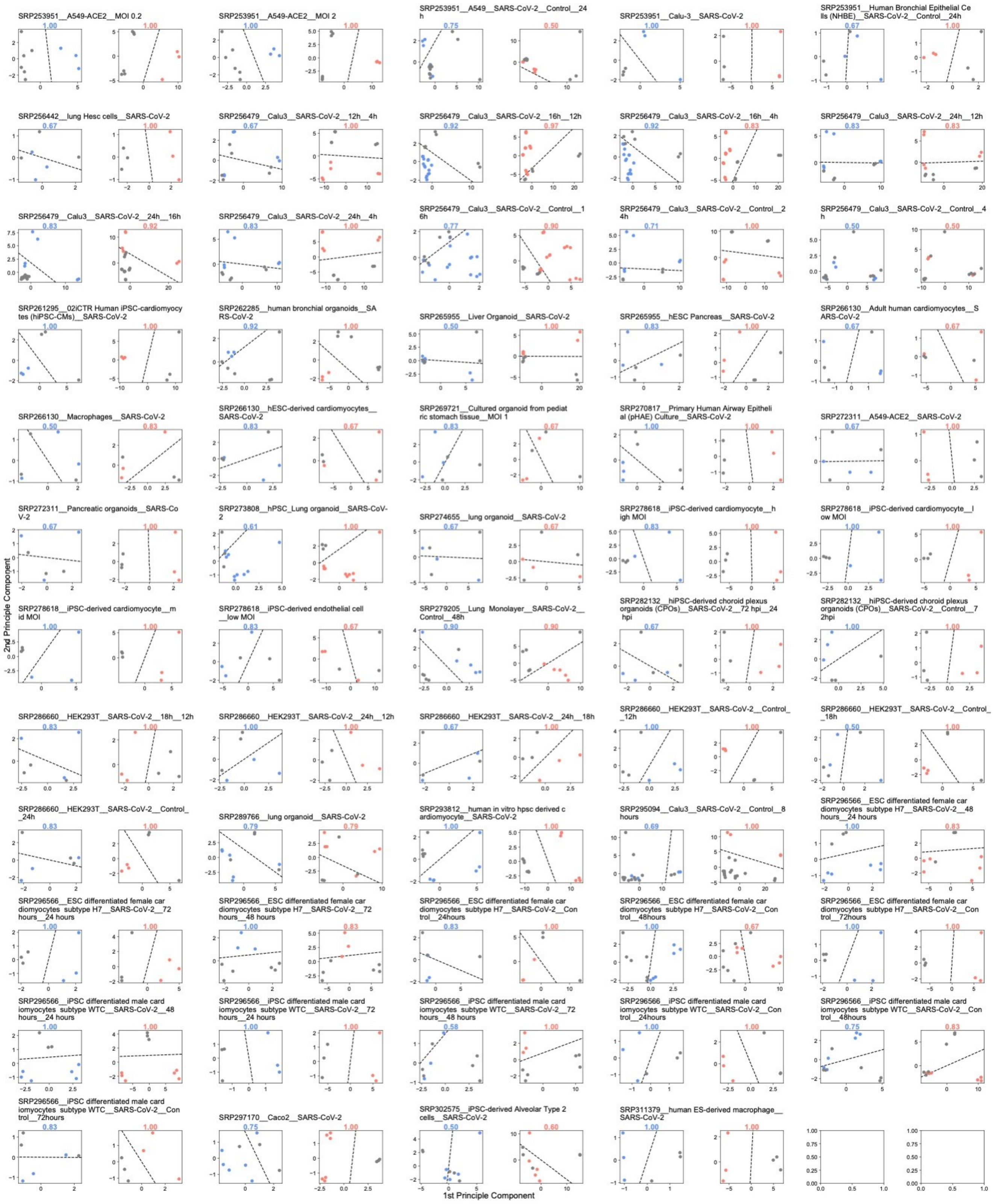
SRP253951__A549-ACE2__MOI 0.2
SRP253951__A549-ACE2__MOI 2
SRP253951__A549__SARS-CoV-2__Control__24h
SRP253951__Calu-3__SARS-CoV-2
SRP253951__Human Bronchial Epithelial Cells (NHBE)__SARS-CoV-2__Control__24h
SRP256442__lung Hesc cells__SARS-CoV-2
SRP256479__Calu3__SARS-CoV-2__12h__4h
SRP256479__Calu3__SARS-CoV-2__16h__12h
SRP256479__Calu3__SARS-CoV-2__16h__4h
SRP256479__Calu3__SARS-CoV-2__24h__12h
SRP256479__Calu3__SARS-CoV-2__24h__16h
SRP256479__Calu3__SARS-CoV-2__24h__4h
SRP256479__Calu3__SARS-CoV-2__Control__16h
SRP256479__Calu3__SARS-CoV-2__Control__24h
SRP256479__Calu3__SARS-CoV-2__Control__4h
SRP261295__02iCTR Human iPSC-cardiomyocytes (hiPSC-CMs)__SARS-CoV-2
SRP262285__human bronchial organoids__SARS-CoV-2
SRP265955__Liver Organoid__SARS-CoV-2
SRP265955__hESC Pancreas__SARS-CoV-2
SRP266130__Adult human cardiomyocytes__SARS-CoV-2
SRP266130__Macrophages__SARS-CoV-2
SRP266130__hESC-derived cardiomyocytes__SARS-CoV-2
SRP269721__Cultured organoid from pediatric stomach tissue__MOI 1
SRP270817__Primary Human Airway Epithelial (pHAE) Culture__SARS-CoV-2
SRP272311__A549-ACE2__SARS-CoV-2
SRP272311__Pancreatic organoids__SARS-CoV-2
SRP273808__hPSC_Lung organoid__SARS-CoV-2
SRP274655__lung organoid__SARS-CoV-2
SRP278618__iPSC-derived cardiomyocyte__high MOI
SRP278618__iPSC-derived cardiomyocyte__low MOI
SRP278618__iPSC-derived cardiomyocyte__mid MOI
SRP278618__iPSC-derived endothelial cell__low MOI
SRP279205__Lung Monolayer__SARS-CoV-2__Control__48h
SRP282132__hiPSC-derived choroid plexus organoids (CPOs)__SARS-CoV-2__72 hpi__24 hpi
SRP282132__hiPSC-derived choroid plexus organoids (CPOs)__SARS-CoV-2__Control__72hpi
SRP286660__HEK293T__SARS-CoV-2__18h__12h
SRP286660__HEK293T__SARS-CoV-2__24h__12h
SRP286660__HEK293T__SARS-CoV-2__24h__18h
SRP286660__HEK293T__SARS-CoV-2__Control__12h
SRP286660__HEK293T__SARS-CoV-2__Control__18h
SRP286660__HEK293T__SARS-CoV-2__Control__24h
SRP289766__lung organoid__SARS-CoV-2
SRP293812__human in vitro hpsc derived cardiomyocyte__SARS-CoV-2
SRP295094__Calu3__SARS-CoV-2__Control__8 hours
SRP296566__ESC differentiated female cardiomyocytes subtype H7__SARS-CoV-2__48 hours__24 hours
SRP296566__ESC differentiated female cardiomyocytes subtype H7__SARS-CoV-2__72 hours__24 hours
SRP296566__ESC differentiated female cardiomyocytes subtype H7__SARS-CoV-2__72 hours__48 hours
SRP296566__ESC differentiated female cardiomyocytes subtype H7__SARS-CoV-2__Control__24hours
SRP296566__ESC differentiated female cardiomyocytes subtype H7__SARS-CoV-2__Control__48hours
SRP296566__ESC differentiated female cardiomyocytes subtype H7__SARS-CoV-2__Control__72hours
SRP296566__iPSC differentiated male cardiomyocytes subtype WTC__SARS-CoV-2__48 hours__24 hours
SRP296566__iPSC differentiated male cardiomyocytes subtype WTC__SARS-CoV-2__72 hours__24 hours
SRP296566__iPSC differentiated male cardiomyocytes subtype WTC__SARS-CoV-2__72 hours__48 hours
SRP296566__iPSC differentiated male cardiomyocytes subtype WTC__SARS-CoV-2__Control__24hours
SRP296566__iPSC differentiated male cardiomyocytes subtype WTC__SARS-CoV-2__Control__48hours
SRP296566__iPSC differentiated male cardiomyocytes subtype WTC__SARS-CoV-2__Control__72hours
SRP297170__Caco2__SARS-CoV-2
SRP302575__iPSC-derived Alveolar Type 2 cells__SARS-CoV-2
SRP311379__human ES-derived macrophage__SARS-CoV-2
2nd Principle Component
1st Principle Component

Figure S6. Verifying the CoV meta-signature v1 with SARS-CoV-2 infection pre-clinical models. A, Violin plot to compare how well infected samples are separated from controls, or different infection time, using the CoV signature genes or random genes. Each point indicates the separation accuracy for a comparison. The p-value was derived from Wilcoxon rank sums test. B, Examples of PCA plots of RNA-seq samples from different comparison groups. Study IDs, model types and treatment settings are denoted in titles. For each comparison, the left PCA plot was derived from a random gene expression matrix of infection/time-point-1 (blue) or control/time-point-2 (grey) samples, while the right PCA plot was based on the CoV signature genes where orange scatters indicate infection/time-point-1 samples. For each PCA plot, a dashed line indicates the boundary between the two categories, and the separation accuracy is labeled above the plot. PCA: principal component analysis. Related to Figure 4.

Table S5. Q-PCR evaluation of IFNB, CXCL10 and IL6 expression fold changes induced by small molecules. Related to Figure 7.

Batch	Treatment	IFNB	CXCL10	IL6
1	DMSO	1.04 ± 0.36	1.18 ± 0.87	1.19 ± 0.79
	IMD-0354	15.49 ± 7.24	9.39 ± 5.23	36.18 ± 9.68
	BX795	0.53 ± 0.17	1.18 ± 0.52	0.67 ± 0.11
	IMD-0354+BX795	1.17 ± 0.30	1.80 ± 1.10	12.24 ± 4.89
2	DMSO	1.00 ± 0.05	1.00 ± 0.02	1.10 ± 0.60
	diABZI	9.49 ± 4.36	24.95 ± 6.77	8.14 ± 2.88

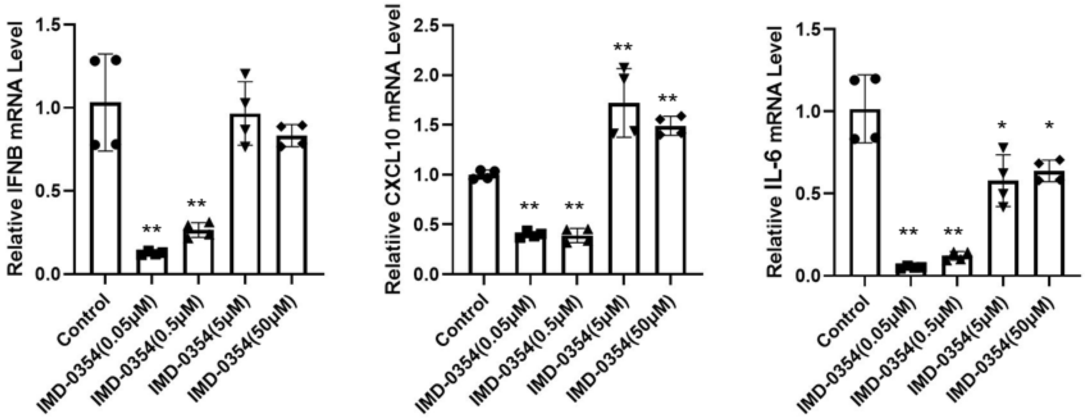

Figure S7. RT-qPCR results on the expression fold change of IFN-β, CXCL10, and IL6 by different concentrations of IMD-0354 compared with DMSO in macrophages derived from Thp-1 cells without viral infection. Error bars denote standard deviations (n=4). **: $p < 0.01$; *: $p < 0.05$; Student's t-tests were performed on each treatment group compared with the control group. Related to Figure 7.

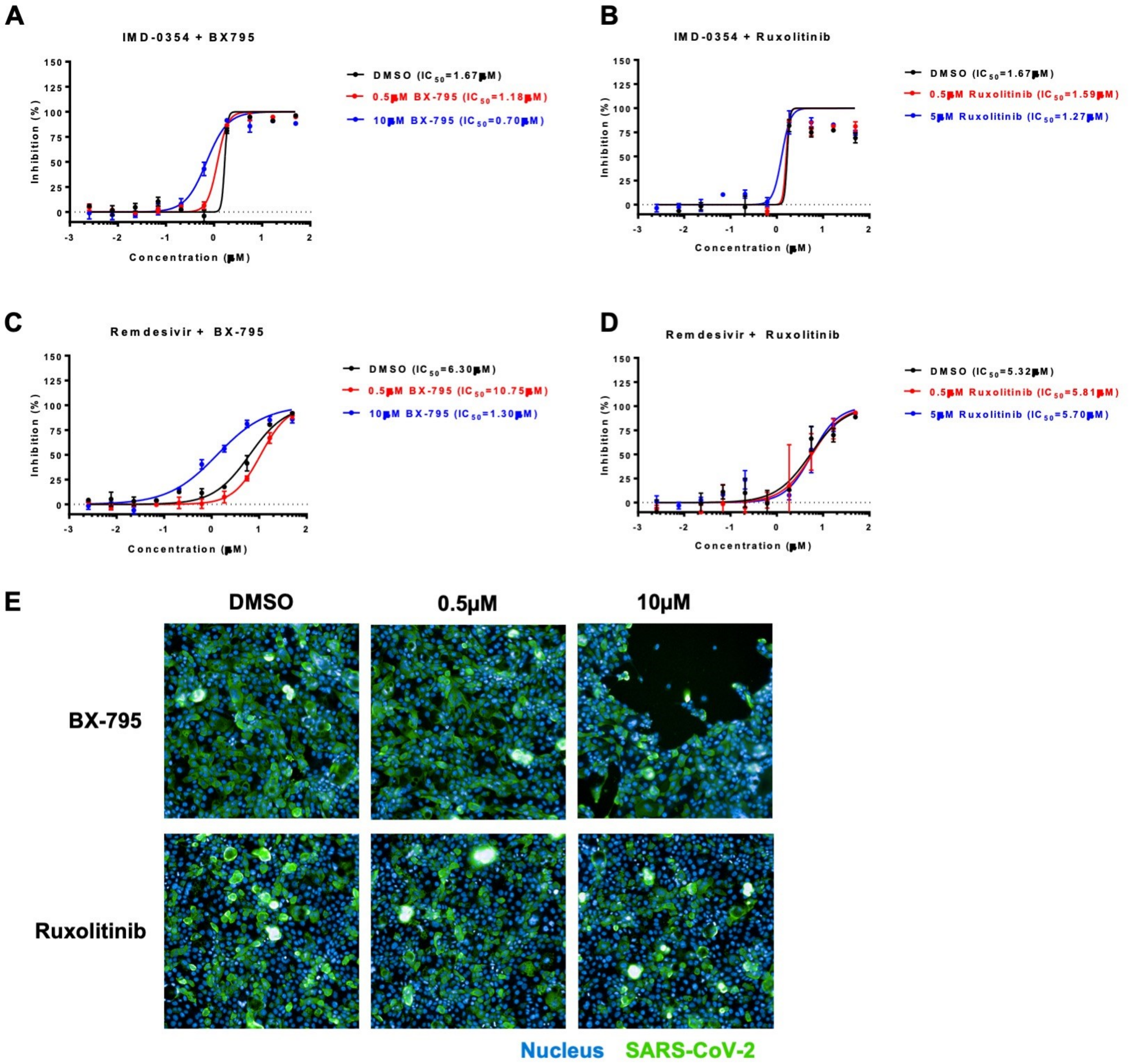

Figure S8. Drug co-treatment and the anti-SARS-CoV-2 dose-response curves (DRCs) in Calu-3 cells. A, The DRC of IMD-0354 given 0.5 or 10 μM of BX795. B, The DRC of IMD-0354 given 0.5 or 5 μM of ruxolitinib. C, The DRC of remdesivir given 0.5 or 10 μM of BX795. D, The DRC of remdesivir given 0.5 or 5 μM of ruxolitinib. IC_{50} and CC_{50} values were measured in duplicates. For all DRC graphs, inhibition of infectivity was quantified by immunofluorescence of SARS-CoV-2 nucleoprotein in Calu-3 cells. E, Calu-3 cell morphology representative images after treated with BX-795 or ruxolitinib. Nucleus is represented in blue and SARS-CoV-2 nucleoprotein is shown in green marks. Treatment of 10μM BX-795 resulted in cell detachment. Related to Figure 7.
